# Supplementary material for: Age-related changes in behavioural and neural variability in a decision-making task
Source: Nat Commun. 2026 Jun 30;17:8156. doi: 10.1038/s41467-026-74227-1 (PMC13458552; doi:10.1038/s41467-026-74227-1)
Supplement: Supplementary file 2 [file 41467_2026_74227_MOESM2_ESM.pdf]

nature portfolio

Corresponding author(s): Fenying Zang, Anne Urai

Last updated by author(s): Apr 30, 2026

# Reporting Summary

Nature Portfolio wishes to improve the reproducibility of the work that we publish. This form provides structure for consistency and transparency in reporting. For further information on Nature Portfolio policies, see our Editorial Policies and the Editorial Policy Checklist.

## Statistics

For all statistical analyses, confirm that the following items are present in the figure legend, table legend, main text, or Methods section.

| n/a | Confirmed | |
|---|---|---|
| ☐ | ☒ | The exact sample size (*n*) for each experimental group/condition, given as a discrete number and unit of measurement |
| ☐ | ☒ | A statement on whether measurements were taken from distinct samples or whether the same sample was measured repeatedly |
| ☐ | ☒ | The statistical test(s) used AND whether they are one- or two-sided<br>*Only common tests should be described solely by name; describe more complex techniques in the Methods section.* |
| ☐ | ☒ | A description of all covariates tested |
| ☒ | ☐ | A description of any assumptions or corrections, such as tests of normality and adjustment for multiple comparisons |
| ☐ | ☒ | A full description of the statistical parameters including central tendency (e.g. means) or other basic estimates (e.g. regression coefficient) AND variation (e.g. standard deviation) or associated estimates of uncertainty (e.g. confidence intervals) |
| ☐ | ☒ | For null hypothesis testing, the test statistic (e.g. *F*, *t*, *r*) with confidence intervals, effect sizes, degrees of freedom and *P* value noted<br>*Give P values as exact values whenever suitable.* |
| ☒ | ☐ | For Bayesian analysis, information on the choice of priors and Markov chain Monte Carlo settings |
| ☐ | ☒ | For hierarchical and complex designs, identification of the appropriate level for tests and full reporting of outcomes |
| ☒ | ☐ | Estimates of effect sizes (e.g. Cohen's *d*, Pearson's *r*), indicating how they were calculated |

*Our web collection on statistics for biologists contains articles on many of the points above.*

## Software and code

Policy information about availability of computer code

| | |
|---|---|
| Data collection | Data were collected using the standardized International Brain Laboratory task and Neuropixels electrophysiology acquisition framework. The electrophysiology recording protocol is described in Appendix 2: IBL protocol: (https://figshare.com/articles/online_resource/Appendix_2_IBL_protocol_for_electrophysiology_recording_using_Neuropixels_probe/19697896/2?file=35882792). No custom data-collection software or algorithm was developed for this study. |
| Data analysis | Data were analyzed using custom Python scripts available at https://github.com/Fenying-Zang/Ageing_behavioral_and_neural_variability. Analyses were run in a reproducible Python environment (https://github.com/int-brain-lab/iblenv). The main directly used software and packages included Python, R, ONE-api, NumPy, pandas, pingouin, ibllib, iblatlas, rpy2, matplotlib and seaborn. The full computational environment, including all package dependencies and version number requirements, is available in the GitHub repository. |

For manuscripts utilizing custom algorithms or software that are central to the research but not yet described in published literature, software must be made available to editors and reviewers. We strongly encourage code deposition in a community repository (e.g. GitHub). See the Nature Portfolio guidelines for submitting code & software for further information.

## Data

Policy information about availability of data

All manuscripts must include a data availability statement. This statement should provide the following information, where applicable:

- Accession codes, unique identifiers, or web links for publicly available datasets
- A description of any restrictions on data availability
- For clinical datasets or third party data, please ensure that the statement adheres to our policy

All data supporting the findings of this study are publicly available and can be accessed through the International Brain Laboratory ONE API; instructions are provided in the IBL documentation (https://docs.internationalbrainlab.org/notebooks_external/data_release_brainwidemap.html) and the study GitHub repository (https://github.com/Fenying-Zang/Ageing_behavioral_and_neural_variability). The intermediate analysis files required to reproduce the figures are available through the same GitHub repository. There are no restrictions on data access.

## Research involving human participants, their data, or biological material

Policy information about studies with human participants or human data. See also policy information about sex, gender (identity/presentation), and sexual orientation and race, ethnicity and racism.

| | |
|---|---|
| Reporting on sex and gender | *Use the terms sex (biological attribute) and gender (shaped by social and cultural circumstances) carefully in order to avoid confusing both terms. Indicate if findings apply to only one sex or gender; describe whether sex and gender were considered in study design; whether sex and/or gender was determined based on self-reporting or assigned and methods used. Provide in the source data disaggregated sex and gender data, where this information has been collected, and if consent has been obtained for sharing of individual-level data; provide overall numbers in this Reporting Summary. Please state if this information has not been collected. Report sex- and gender-based analyses where performed, justify reasons for lack of sex- and gender-based analysis.* |
| Reporting on race, ethnicity, or other socially relevant groupings | *Please specify the socially constructed or socially relevant categorization variable(s) used in your manuscript and explain why they were used. Please note that such variables should not be used as proxies for other socially constructed/relevant variables (for example, race or ethnicity should not be used as a proxy for socioeconomic status). Provide clear definitions of the relevant terms used, how they were provided (by the participants/respondents, the researchers, or third parties), and the method(s) used to classify people into the different categories (e.g. self-report, census or administrative data, social media data, etc.) Please provide details about how you controlled for confounding variables in your analyses.* |
| Population characteristics | *Describe the covariate-relevant population characteristics of the human research participants (e.g. age, genotypic information, past and current diagnosis and treatment categories). If you filled out the behavioural & social sciences study design questions and have nothing to add here, write "See above."* |
| Recruitment | *Describe how participants were recruited. Outline any potential self-selection bias or other biases that may be present and how these are likely to impact results.* |
| Ethics oversight | *Identify the organization(s) that approved the study protocol.* |

Note that full information on the approval of the study protocol must also be provided in the manuscript.

# Field-specific reporting

Please select the one below that is the best fit for your research. If you are not sure, read the appropriate sections before making your selection.

[x] Life sciences [ ] Behavioural & social sciences [ ] Ecological, evolutionary & environmental sciences

For a reference copy of the document with all sections, see nature.com/documents/nr-reporting-summary-flat.pdf

# Life sciences study design

All studies must disclose on these points even when the disclosure is negative.

| | |
|---|---|
| Sample size | No statistical method was used to predetermine sample size. Sample sizes for the public dataset (younger mice) were determined by the availability of animals in the IBL brain-wide map paper. Sample sizes for the newly recorded dataset (older mice) were determined by experimental and practical constraints during data acquisition. All animals, sessions, probes and neurons passing the pre-established inclusion and quality-control criteria were included. The resulting dataset included 149 mice, 367 sessions, 503 insertions and 18,755 good neurons across 16 brain regions. |
| Data exclusions | Data exclusions followed pre-established session-, insertion-, trial- and neuron-level quality-control criteria, primarily based on established IBL and RIGOR criteria, with additional single-neuron stability requirements for Fano Factor analyses. Exclusions were based on behavioral performance, hardware quality control, recording stability, alignment, spiking sorter version, missing trial events, RT range, single-unit quality control, firing rate, presence ratio and ROI inclusion. Full criteria are described in the Methods. |
| Replication | Reproducibility was supported by the use of standardized IBL task and Neuropixels recording protocols, established IBL quality-control |

procedures. Reproducibility of the task and the electrophysiological measurements are extensively discussed in https://elifesciences.org/articles/63711 and https://elifesciences.org/articles/100840. No independent replication cohort was collected specifically for this study.

| | |
|---|---|
| Randomization | Animals were not randomized into age groups because age was the biological variable of interest. No treatment allocation was performed. |
| Blinding | Since animals were co-housed with littermates, we could not collect data blinded to animals' age. |

# Reporting for specific materials, systems and methods

We require information from authors about some types of materials, experimental systems and methods used in many studies. Here, indicate whether each material, system or method listed is relevant to your study. If you are not sure if a list item applies to your research, read the appropriate section before selecting a response.

## Materials & experimental systems

| n/a | Involved in the study | |
|---|---|---|
| ☒ | ☐ | Antibodies |
| ☒ | ☐ | Eukaryotic cell lines |
| ☒ | ☐ | Palaeontology and archaeology |
| ☐ | ☒ | Animals and other organisms |
| ☒ | ☐ | Clinical data |
| ☒ | ☐ | Dual use research of concern |
| ☒ | ☐ | Plants |

## Methods

| n/a | Involved in the study | |
|---|---|---|
| ☒ | ☐ | ChIP-seq |
| ☒ | ☐ | Flow cytometry |
| ☒ | ☐ | MRI-based neuroimaging |

## Animals and other research organisms

Policy information about studies involving animals; ARRIVE guidelines recommended for reporting animal research, and Sex and Gender in Research

| | |
|---|---|
| Laboratory animals | the C57BL/6 laboratory mice |
| Wild animals | n/a |
| Reporting on sex | both sexes were included and reported |
| Field-collected samples | n/a |
| Ethics oversight | For the public dataset: All procedures and experiments were carried out in accordance with the local laws and following approval by the relevant institutions. The relevant institutions were: the Animal Welfare Ethical Review Body of University College London; the Institutional Animal Care and Use Committees of Cold Spring Harbor Laboratory, Princeton University, University of Washington, University of California at Berkeley and University of California at Los Angeles; the University Animal Welfare Committee of New York University; and the Portuguese Veterinary General Board.<br><br>For the newly collected dataset: All procedures and experiments were carried out in accordance with the local laws and following approval by the relevant institutions. The relevant institutions were: the Institutional Animal Care and Use Committees of Cold Spring Harbor Laboratory; the Animal Welfare Ethical Review Body of University College London |

Note that full information on the approval of the study protocol must also be provided in the manuscript.

## Plants

| | |
|---|---|
| Seed stocks | *Report on the source of all seed stocks or other plant material used. If applicable, state the seed stock centre and catalogue number. If plant specimens were collected from the field, describe the collection location, date and sampling procedures.* |
| Novel plant genotypes | *Describe the methods by which all novel plant genotypes were produced. This includes those generated by transgenic approaches, gene editing, chemical/radiation-based mutagenesis and hybridization. For transgenic lines, describe the transformation method, the number of independent lines analyzed and the generation upon which experiments were performed. For gene-edited lines, describe the editor used, the endogenous sequence targeted for editing, the targeting guide RNA sequence (if applicable) and how the editor was applied.* |
| Authentication | *Describe any authentication procedures for each seed stock used or novel genotype generated. Describe any experiments used to assess the effect of a mutation and, where applicable, how potential secondary effects (e.g. second site T-DNA insertions, mosiacism, off-target gene editing) were examined.* |
