# Supplementary material for: Age-related changes in behavioural and neural variability in a decision-making task
Source: Nat Commun. 2026 Jun 30;17:8156. doi: 10.1038/s41467-026-74227-1 (PMC13458552; doi:10.1038/s41467-026-74227-1)
Supplement: Supplementary file 3 [file 41467_2026_74227_MOESM3_ESM.pdf]

nature portfolio

Peer Review File

Age-related changes in behavioral and neural variability in a decision-making task

Corresponding Author: Ms Fenying Zang

This file contains all reviewer reports in order by version, followed by all author rebuttals in order by version.

Version 0:

Reviewer comments:

Reviewer #1

(Remarks to the Author)
In this study the authors investigate the effects of mouse age on behavior in the IBL decision making task, and on neural coding during the task. The study combines an existing large scale dataset with supplementary behavior and recordings from older mice. The dataset is therefore large both in subjects and recorded neurons. The effects of aging on brain function are important and the study contributes to the existing literature by replicating in mice a number of findings observed in other species, and provides a systematic survey across brain regions. The study is generally well written and the results are clearly presented. I commend the authors for releasing their code and using a large scale public dataset. I have a number of concerns that impact interpretation of the results, as well as a few statistical concerns.

Major concerns
1. This study analyzes age from N=130 mice from IBL et al 2023 (first dataset), as well as N=19 older mice recorded for this study (new dataset). Are there differences between the two datasets, other than age, such as changes in breeding, rearing, housing, or vendor? My understanding is that the first dataset was collected across many labs, so it’s possible that any variation between the first dataset and the new dataset is smaller than the variation within the first dataset itself. Please comment on any potential differences between the collection of the new dataset and the original dataset. Additionally, a supplemental figure showing the main age effects of Figure 1 using only mice from the original dataset would be valuable to rule out any cohort effects.

2a. The source of age variability in the first dataset is not clear. Were some mice older during recording because of poor learning, or variability in operations of breeding, training, and recording? Please comment on the source of age variability in the first dataset.

2b. I am particularly concerned about ruling out an alternative interpretation that the behavioral and neural effects of age in this study are due to either slow task learning, or longer training duration training. Looking at supplemental Figure 1C it appears that older mice also took longer to learn. My understanding is that age in the x-axis of Fig S1C (and throughout the paper) is age at time of recording. This makes it hard to interpret whether the mice learned slower because they were older, or whether the mice were older at the time of recording because they learned slower. Please provide a control analysis that address the confound between slower learning and age. For example, you could potentially use age at time of training start rather than age at time of recording. Or, you could subsample mice such that the number of training days is comparable between younger and older mice.

3. The authors report that the main statistical method used in the paper is a generalized linear model. However, examining the methods section, it appears the authors actually used a general linear model. This terminology is commonly confused in the literature, so I think it’s important to clarify. A general linear model is linear Y ~ WX with residual errors assumed to be gaussian distributed, whereas a generalized linear model is nonlinear and “generalizes” to non-gaussian noise models Y ~ f(WX). My apologies if I misunderstand the approach used. In most of the plots that use a regression, the y-axis is strictly positive, and in some plots the distribution appears to have a long tail towards large values. This makes me think that a general linear model with a gaussian noise model may be in-appropriate for this data. This issue seems the most pronounced in the insets of Fig 1C.

Please clarify the statistical method used - general linear model or generalized linear model. If a general linear model was

used please comment on whether the residuals were approximately normally distributed or use an alternative approach to deal with non-normal data. If a generalized linear model was used, please report the link function and noise model used.

4. It is not clear to me whether the regression analyses in Figure 3 and 4 were computed on a cell by cell basis or a probe insertion by insertion basis. Was each of the neural metrics (firing rate, contrast modulation slope, FF, etc) computed for each cell, or averaged for each insertion? Computing these metrics on a cell by cell basis is the standard approach and most interpretable, but the plots in Figure 3/4 show the regression line over the data grouped by probe insertion. Further the methods section does not clarify the approach used, since neural yield must be computed on a insertion basis but neural yield is listed alongside other neural metrics. Please clarify how the regression analyses were computed. If analyses were computed on metrics averaged for each insertion, please justify this approach.

Minor concerns

5. It is not clear to me how to interpret mouse age against literature reports from other species - the authors cite studies in humans, monkeys, and cats. I would appreciate a brief review of how results in the literature have defined young/old groups in other species. I appreciate the citation of Cottam et al, 2025 which roughly maps mouse age 3-20 months to human 23-70, this helps provide meaningful context. Adding this reference in the introduction would help clarify the focus of this study - middle age of life and not adolescence or elderly populations.

6. When did mouse behavior and neural recordings happen with respect to each mouses' light cycle? Is it possible that differences in chronotype with age interact with the findings of this paper?

7. On pg 4, "older animals do not strongly differ in the degree…(Figure 1-S3; Findling et al, 2023). Please clarify the results from this study versus those in the reference Findling et al 2023.

8. On pg 4 "Older mice did not respond more slowly, but were more variable in their RTs". This implies that older mice sometimes have faster reaction times than younger mice. This appears to be true in Figure 1G, the older mice have more probability mass in the first three time bins. My understanding is that trials with RTs < 80ms were excluded. Were young mice more impulsive, and thus more heavily impacted by this 80ms cutoff?

9. Does the regression on the inset of Fig 1D use all contrast levels? Would you get the same result if you only looked at contrast = 0 trials? I'm nitpicking here along with concern #8 because I'm interested in the possibility that older mice use cognitive effort in a more dynamic way - longer focus/RT on hard trials, and less on easier trials.

10. Was there any difference in premature responses, or other violation type trials/responses with age?

11. How was the cutoff of 2s for RT determined? It appears the young mice have a longer tail in Fig 1G, so this cutoff may influence the behavioral conclusions of Fig 1.

12. Does the confidence interval in Fig 3A take into account the hierarchical nature of the neural data? My understanding is that when computing p-values all cells from each session were shuffled together, it's not clear if a similar approach was used in Figure 3A.

13. Fig 3H, this panel appears to not be referenced anywhere in the text.

14. Fig 4A. It appears that the FF increases for older mice with stimulus onset, peaking around 100ms compared to around 0ms for younger mice. What is the source of this increase in variability?

15. Please add line numbers to the text in order to help with reviewing.

(Remarks on code availability)

Reviewer #2

(Remarks to the Author)

This study provides comprehensive and standardized evidence of how aging affects behavior and neural activity in mice. The authors carefully investigated how choices during the International Brain Laboratory (IBL) task were modulated by aging. They found that older mice showed reduced choice accuracy and more variable reaction times compared to younger mice. The authors then examined neuronal activity during the IBL task and found that aging increased firing rates and post-stimulus neural variability in various brain regions. In particular, the authors focused on the increase in neural variability caused by aging.

Although this study does not include many results to support its conclusions, the relationship among decision-making, neural activity, and aging was not well understood in previous studies. This work provides essential findings on how aging affects the neural circuits involved in decision-making. I mainly have three major comments regarding the validation of (1) neural activity, (2) behavior, and (3) conceptual advances from previous literature in rodents.

Major comments

1. As reported in the author's previous work (Musall et al., Nat. Neurosci., 2019), neural activity is modulated by movement.

This study showed that aging increased the variability of reaction times and neural activity after visual inputs. Was the modulation of activity merely a consequence of behavioral variability, or was it an effect of aging? The IBL behavioral setup includes a video camera; therefore, the authors can quantify and separate the neural modulation due to behavioral variability from that due to aging.

2. In the previous IBL study (Figure 2, International Brain Laboratory et al., eLife, 2021), the learning rates of mice depended on the laboratory. In this study, the newly added data on older mice comprise the main results. It is important to determine whether the aging effects observed in choice behavior during the electrophysiological and learning phases were not the result of laboratory differences. It is also important to analyze whether the behavioral differences reported here did not depend on when the data were collected, as the older-mice data were relatively recent.

3. This study does not sufficiently cite previous rodent studies on aging. It has already been reported that aging modulates neural activity in rats and mice (Maziar et al., Cereb Cortex, 2023; Simkin et al., J Neurosci, 2015; Fischer et al., Cell Mol Life Sci, 2021; Randall et al., Neurobiol Aging, 2012). Effects of aging have also been reported in the field of Alzheimer's disease (Anastacio et al., Transl Psychiatry, 2022; Sosulina et al., J Neurochem, 2021; Wang et al., Behav Brain Res, 2017). Please summarize rodent studies on aging in the Introduction and Discussion to clarify the conceptual advances of the present study.

Minor comments

4. Page 4, paragraph 1 (Figure 1c). Older mice showed slightly worse performance both on the day they met this criterion and during their first Neuropixels recording session (Figure 1-S1d,f).

I was not sure how the behaviors between young and old mice were different on the day they met the training criteria. As far as I understand, the criteria were the same for all mice, so there should be no behavioral difference in the criteria (e.g. performance on easy trials).

Did the choice differences between young and old mice arise from (1) performance within the criteria, (2) performance outside the criteria, or (3) differences in how the criteria was achieved?

5. Page 4, paragraph 4.

To validate variability, how did the authors decide to use different measurements for behavior (coefficient of variation, CV) and for neural activity (Fano factor)?

6. Page 6, paragraph 1. Overall neural yield was slightly reduced in older mice.

It is interesting to observe that neural yield depends on age. It would be beneficial to provide a hypothesis to explain this observation.

7. Page 7, paragraph 1. Ageing increased firing rates globally.

Related to major comment 3: please include that increased firing rates have also been observed in rodents.

8. Page 8, Figure 3h.

I might be mistaken, but the range of color bar β(age) in Figure 3h appears larger compared to other figures. Does this imply that some brain regions (e.g. visual cortex) showed reduced contrast modulation, while other regions (e.g., hippocampus) showed increased modulation with aging? If so, please revise the last paragraph of page 7.

9. Page 10, last paragraph. By contrast, for Fano Factor quenching we found strong evidence for no effect of training time,

(1) In the Methods section (Page 19), please add how the authors analyzed βtraining.

(2) This result implies that the training time and mouse age were different, although they were correlated. To clarify this paragraph, it would be worth developing a GLM that includes both βtraining and βage.

10. (Optional) Page 13, paragraph 3. Pairwise noise correlations.

As the authors write in the Discussion, changes in post-stimulus neural variability with aging may be related to changes in noise correlations (e.g., Miura et al, 2012). Given the rich population dataset of neural activity, the authors could test whether the results in this study are also related to changes in noise correlations during decision making. This is only a minor, optional suggestion.

(Remarks on code availability)

Version 1:

Reviewer comments:

Reviewer #1

(Remarks to the Author)

The authors have responded to all my concerns with detailed and thoughtful analyses. I now support publication. I thank the reviewers for the careful consideration of the issues raised.

(Remarks on code availability)

Reviewer #2

(Remarks to the Author)
I appreciate that the authors put a great effort into analyzing the movie data and generating additional figures to support their conclusions. The authors have addressed most of my previous comments. However, there is one remaining comment (and one minor change) that should be considered.

1. My previous comment 1 asked the following: "Was the modulation of activity merely a consequence of behavioral variability, or was it an effect of aging?" The authors mainly analyzed the "median" movement speed, which corresponded to Figure 1d. Although it is important to focus on the "median" speeds, I would suggest that the authors also consider the effects of behavioral "variability" on neural activity.
In Figure 1e and f, the authors analyzed reaction-time variability using the coefficient of variation (CV). How did the CV (or other measures of variability) of the DLC movements of the paw and nose-tip at pre- and post-stimulus in Figure 4 S6 correlate with aging?
In Table S3, in addition to using the median speeds, the authors could also use the CV of those speeds as a control to investigate whether the modulation of neural activity was merely a consequence of behavioral "variability" or an effect of aging. By introducing movement CVs, the authors could also test the relationship between the change in movement variability between pre- and post-stimulus periods and Fano factor quenching in neurons. Another possible approach would be for the authors to subsample sessions and analyze neural activity only from sessions with similar movement variability but different ages.

Minor
Caption of Figure 4 S6: median ± 95% CI.
I think "CI" appears here for the first time. Please write it as "confidence interval".

(Remarks on code availability)
The code is well organized. It provides a README file with enough instructions.

Version 2:

Reviewer comments:

Reviewer #2

(Remarks to the Author)
I would like to thank the authors for their detailed response and the efforts made to revise the manuscript. The authors have addressed all my comments satisfactorily.

(Remarks on code availability)

Open Access This Peer Review File is licensed under a Creative Commons Attribution 4.0 International License, which permits use, sharing, adaptation, distribution and reproduction in any medium or format, as long as you give appropriate credit to the original author(s) and the source, provide a link to the Creative Commons license, and indicate if changes were made.
In cases where reviewers are anonymous, credit should be given to 'Anonymous Referee' and the source.
The images or other third party material in this Peer Review File are included in the article's Creative Commons license, unless indicated otherwise in a credit line to the material. If material is not included in the article's Creative Commons license and your intended use is not permitted by statutory regulation or exceeds the permitted use, you will need to obtain permission directly from the copyright holder.
To view a copy of this license, visit https://creativecommons.org/licenses/by/4.0/

Dear editor and reviewers,

We thank the reviewers for their careful reading of our manuscript and for the constructive feedback. We have carefully addressed each of the reviewers' concerns by performing a number of additional analyses and revising the manuscript accordingly.

The main changes in the manuscript include:

1. **Training duration analyses and controls**
    a. We performed a variance-decomposition to clarify sources contributing to age variability (Figure 1-S2; a question raised by R1.2a).
    b. We re-evaluated learning speed using age at training start (rather than age at recording) and updated the presentation and interpretation accordingly (Figure 1-S1; R1.2b).
    c. We fit extended models including both age and training-duration variables to assess how training duration influences behavioral and neural age effects (Table S2; mainly R1.2b and R2.9).
2. **Movement analyses and controls**
    a. We added new analyses to quantify video-derived movement measures and assess age-related changes in movement (Figure 4-S6; R2.1).
    b. We incorporated movement covariates into extended models to test whether the observed age effects persist after accounting for movement (Table S3; R2.1).
3. **Manuscript organization and supplementary materials**
    a. We added a new subsection at the end of the Results (p. 11-12) to summarize these additional robustness analyses and their implications for interpretation.
    b. We substantially expanded the Supplementary Information with 5 additional figures and 3 tables documenting the new analyses.

We believe these additions and revisions considerably strengthen the paper and provide a more rigorous basis for the conclusions. We provide a detailed point-by-point response below (replies in blue), with references to the revised manuscript and supplementary materials.

We hope you will now find our manuscript suitable for publication in *Nature Communications*.

On behalf of all authors,
Fenying Zang and Anne Urai

Point-by-point reply to reviewer #1

In this study the authors investigate the effects of mouse age on behavior in the IBL decision making task, and on neural coding during the task. The study combines an existing large scale dataset with supplementary behavior and recordings from older mice. The dataset is therefore large both in subjects and recorded neurons. The effects of aging on brain function are important and the study contributes to the existing literature by replicating in mice a number of findings observed in other species, and provides a systematic survey across brain regions. The study is generally well written and the results are clearly presented. I commend the authors for releasing their code and using a large scale public dataset. I have a number of concerns that impact interpretation of the results, as well as a few statistical concerns.

We thank Reviewer 1 for the thoughtful evaluation and the constructive comments. We appreciate the positive assessment of the study and the recognition of our commitment to open and reproducible research. We have addressed each concern point-by-point below and indicate the corresponding revisions in the manuscript.

Major comments

R1.1. This study analyzes age from N=130 mice from IBL et al 2023 (first dataset), as well as N=19 older mice recorded for this study (new dataset). Are there differences between the two datasets, other than age, such as changes in breeding, rearing, housing, or vendor? My understanding is that the first dataset was collected across many labs, so it's possible that any variation between the first dataset and the new dataset is smaller than the variation within the first dataset itself. Please comment on any potential differences between the collection of the new dataset and the original dataset. Additionally, a supplemental figure showing the main age effects of Figure 1 using only mice from the original dataset would be valuable to rule out any cohort effects.

We thank the reviewer for raising this important point. Both the original dataset and the new recordings were collected within the same International Brain Laboratory (IBL) framework for standardized behaviour and electrophysiology, using the same decision-making task, training pipeline, hardware and spike-sorting and quality-control procedures (International Brain Laboratory, 2020, 2022a; International Brain Laboratory, Benson, et al., 2025). All mice were C57BL/6, obtained from either Jackson Laboratory or Charles River, and underwent the same IBL training protocol before electrophysiological recordings.

As detailed in the original IBL paper, housing and husbandry followed common guidelines across laboratories: mice were kept on a 12:12 h light–dark cycle (normal or inverted, depending on the laboratory) with food and water available ad libitum outside behavioural training. Other environmental variables such as temperature, humidity and environmental sound were not strictly standardized but were routinely documented and monitored across sites (International Brain Laboratory, Benson, et al., 2025). Importantly, the IBL Reproducible Ephys study reported that laboratory identity could not be decoded from electrophysiological features

under the standardized IBL pipeline, indicating high cross-lab reproducibility and suggesting that any remaining lab-to-lab differences are unlikely to dominate the observed variation (International Brain Laboratory, Banga, et al., 2025).

Regarding the new recordings, most were obtained in the Churchland lab, which also contributed extensively to the original IBL brain-wide map dataset. Two mice were additionally contributed by the Mrsic-Flogel lab.

In the combined sample, mice from different age ranges are combined across labs, and Churchland-lab mice span a broad range of ages in both the original and new datasets (Figure R1).

To address potential effects of these two subsets of data, we repeated the main analyses of Figure 1 in two ways. First, as you suggested, we subsampled the data to include only mice from the original IBL brain-wide map dataset. The age-related effects on response time variability remained robust (Figure R2).

As a second control analysis, we were even more conservative to rule out any effects of the lab. To this end, we repeated the analyses using only sessions recorded in the Churchland lab (Figure R3). This considerably reduced our sample size (63 sessions). Importantly, while this lower power reduces the statistical evidence (Bayes Factors), we observe effects of ageing in the same direction.

Together, we believe these control analyses considerably strengthen our ability to focus on ageing as a dominant cross-animal factor driving behavioral variability, beyond lab-to-lab differences. This is in line with our behavioral and neural protocols, which were stringently designed to ensure lab-to-lab reproducibility in both expert behavior and neural recordings (International Brain Laboratory et al., 2021, 2025).

We now summarize this point in the revised manuscript **(**Results, p. 5, lines 6-9**)**:

> “This result was robust to various control analyses: other measures of variability (Figure 1-S5a) and for a definition of RT that captures the first movement initiation, rather than movement completion (Figure 1-S5b), and differences between dataset sources or lab-specific environmental factors (Figure 1-S6, S7).”

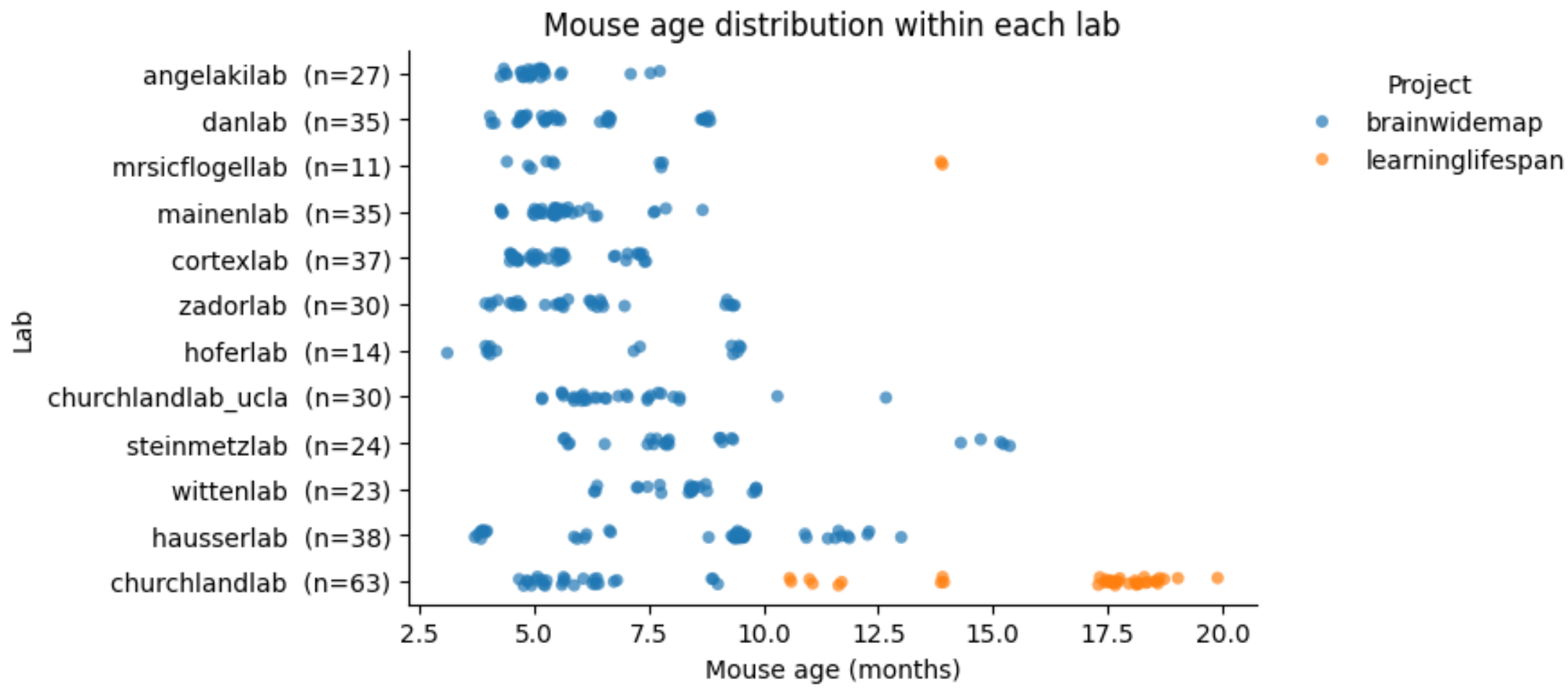

Figure R1. Distribution of mouse age across laboratories and datasets. Each point represents one mouse, plotted by age at recording and grouped by laboratory. Colors indicate dataset (original IBL dataset “brain-wide map” vs. new dataset “lifespan”). Mice of different ages are interleaved across labs, and Churchland-lab mice span a broad age range in both datasets.

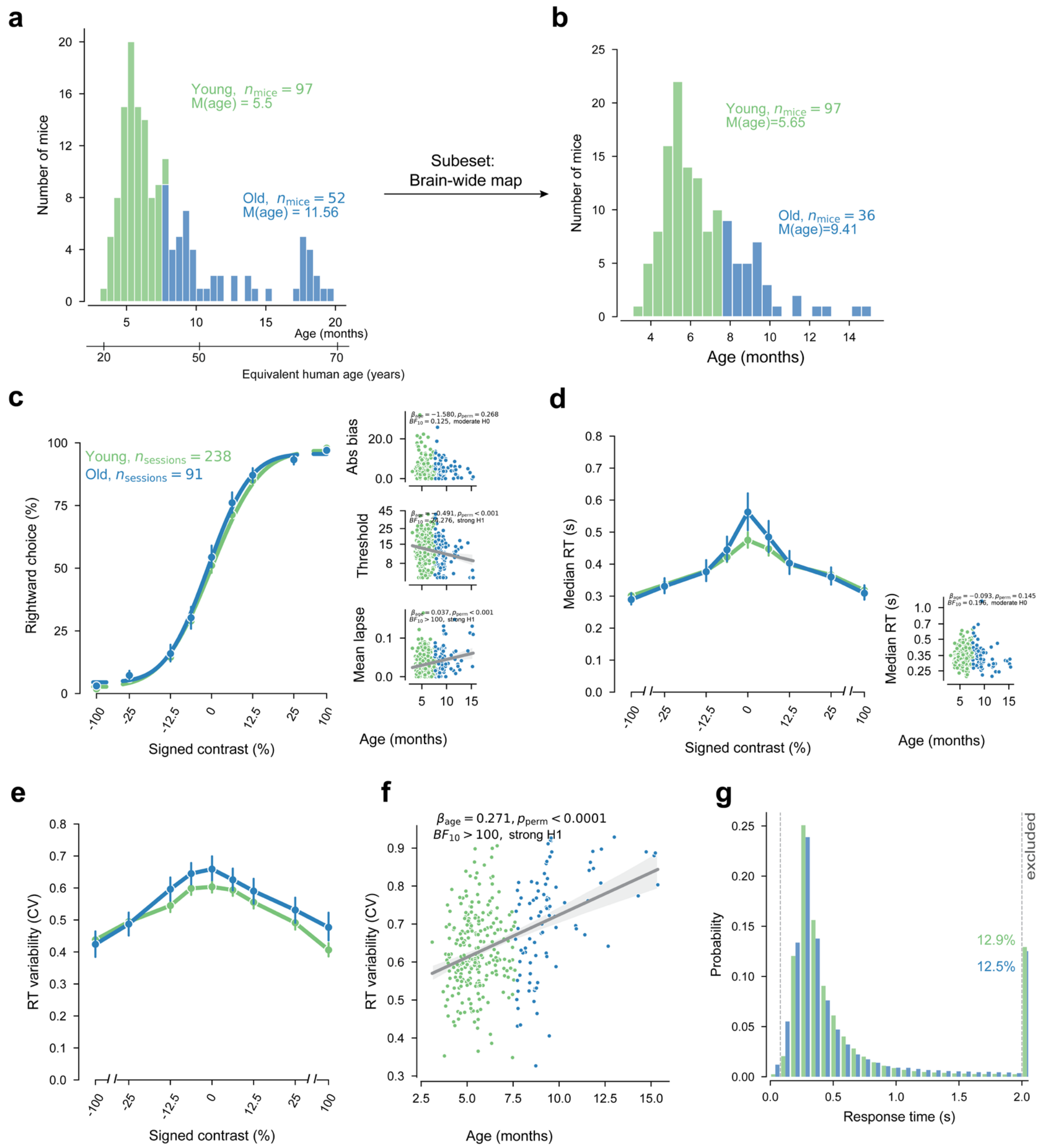

Figure R2 (Figure 1 S6). Replication of Figure 1 using only mice from the original IBL "brain-wide map" dataset. Age-related effects on median RT and RT variability are similar in direction and magnitude to those obtained in the full dataset.

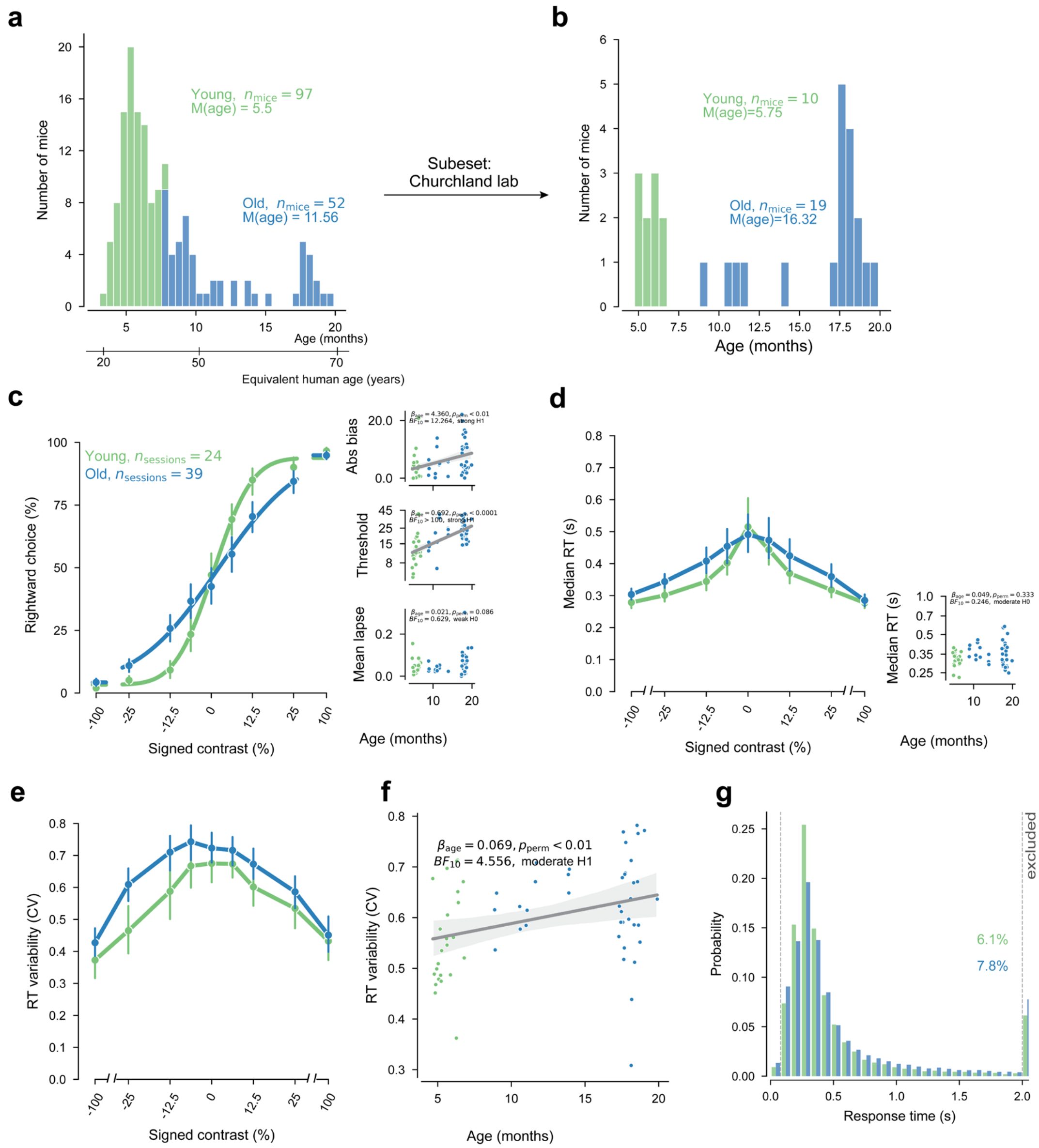

Figure R3 (Figure 1 S7). Replication of Figure 1 using only mice recorded in the Churchland lab.

R1.2a. The source of age variability in the first dataset is not clear. Were some mice older during recording because of poor learning, or variability in operations of breeding, training, and recording? Please comment on the source of age variability in the first dataset.

We agree that clarifying the source of age variability in the IBL datasets is important. As you say, variability in age at recording arises from three factors: **(1) differences in age at the start of behavioural training, (2) differences in the training duration (number of training days), and (3) differences in non-training days between training start and recording (e.g., scheduling or other experimental/logistical constraints).**

Figure R4 visualizes this relationship for the combined datasets (one point per mouse): the x-axis shows age at training start, and the y-axis shows age at first recording. The diagonal (y = x) is the theoretical lower bound of zero elapsed time; the vertical distance from this diagonal therefore reflects the total elapsed time between training onset and the first recording (training duration + non-training gap). Across laboratories, mice occupy a broad range of training start ages and elapsed times, indicating that age variability is not attributable to a single narrow source.

To further characterize these sources, we plotted the distributions of the three components on the same scale (Figure R5, left). We then quantified their relative contributions to between-mouse variability in age at recording using an order-invariant incremental explained-variance analysis (average ΔR^2 across all predictor orderings in an OLS model; Figure R5, right). This attribution indicated that variability in age at training start accounted for **~69%** of the attributed variance in recording age, non-training gaps for ~23%, and training duration for **~8%**. Together, these results show that recording-age variability is largely driven by when mice began training, with smaller contributions from non-training gaps and training duration.

We mentioned it briefly in Results (p. 4, lines 15-18):

> “Although animals that took longer to learn were then older by the time they moved on to neural recordings, the main source of age variability at recording time arises from starting the training program at a later age (Figure 1-S2).”

We also added a brief description of these sources in the Methods (p. 16-17, lines 38-1):

> “Age was calculated on the day of electrophysiological recording. Across mice, age at recording varied due to differences in (1) age at the start of behavioural training, (2) training duration, and (3) non-training intervals between training start and the first recording (e.g., experimental or logistical constraints). A decomposition of these sources indicates that variability is dominated by differences in age at training start (Figure 1-S2).”

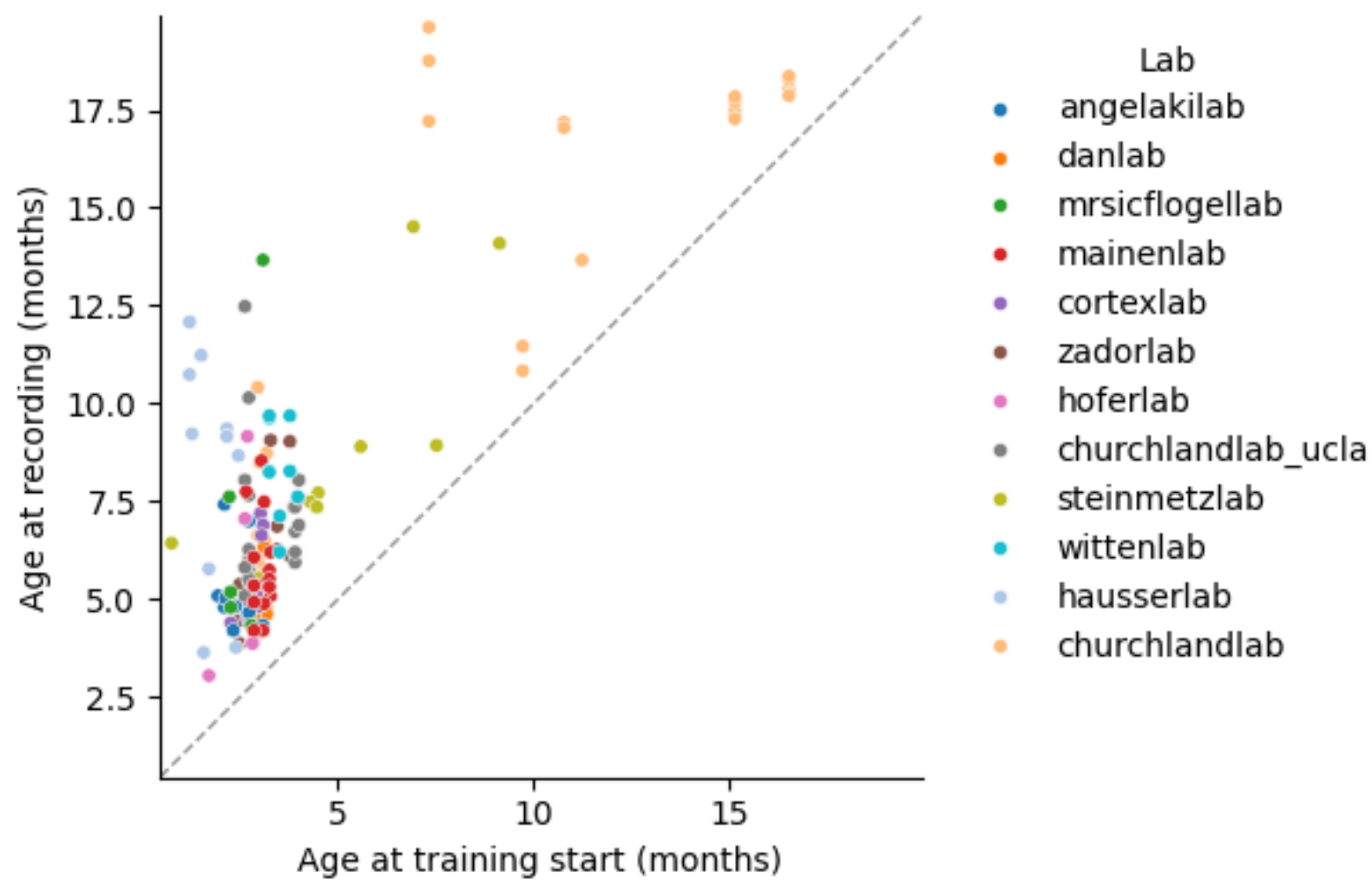

Fig R4. Age at training start vs. age at recording. Scatter plot showing one point per mouse for the combined "brain-wide map" and "lifespan" datasets. The x-axis indicates age at behavioural training start and the y-axis indicates age at the recording. The diagonal (y = x) is the theoretical lower bound corresponding to zero elapsed time between training start and recording. The vertical distance above the diagonal reflects the elapsed time from training onset to the recording session (training duration + non-training gap). Points are coloured by laboratory.

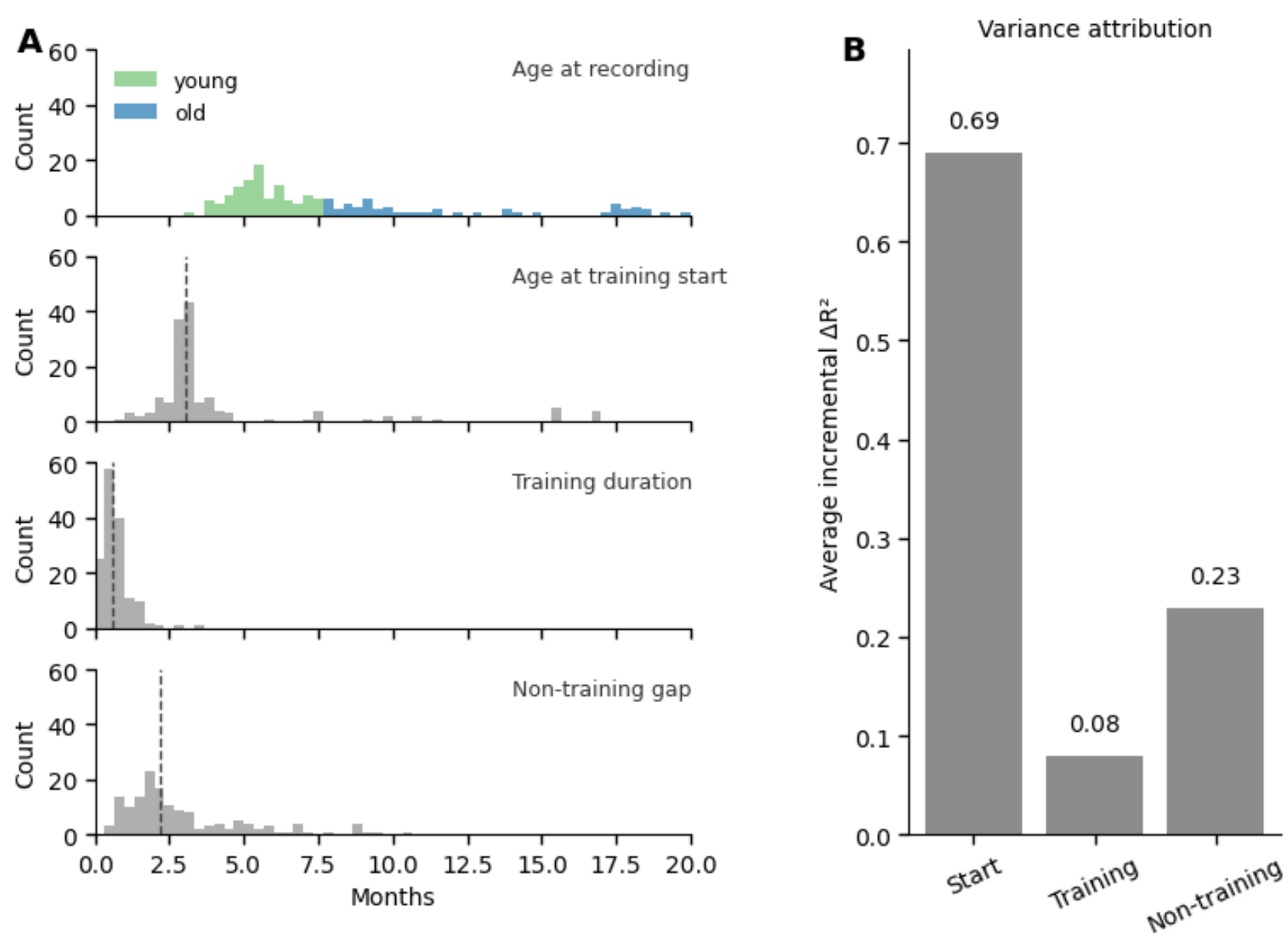

Fig R5 (Figure 1 S2). Components contributing to variability in age at recording. (a) Distributions (in months) of age at recording and the three components used to decompose age at recording: age at training start, training duration (number of training days), and the non-training gap between training start and the recording. Dash lines indicate the median. **(b)** Order-invariant variance attribution using average incremental ΔR^2 across all predictor orderings in an OLS model.

R1.2b. I am particularly concerned about ruling out an alternative interpretation that the behavioral and neural effects of age in this study are due to either slow task learning, or longer

training duration training. Looking at supplemental Figure 1C it appears that older mice also took longer to learn. My understanding is that age in the x-axis of Fig S1C (and throughout the paper) is age at time of recording. This makes it hard to interpret whether the mice learned slower because they were older, or whether the mice were older at the time of recording because they learned slower. Please provide a control analysis that address the confound between slower learning and age. For example, you could potentially use age at time of training start rather than age at time of recording. Or, you could subsample mice such that the number of training days is comparable between younger and older mice.

Thank you for raising this important point. We agree that age at recording can be confounded with training duration, and we addressed this in two complementary ways. This has led us to change our conclusion of the learning-speed result, as described below.

First, we re-analysed learning speed using **age at training start** rather than age at recording. We computed training duration (number of training days to reach the standardized behavioral criteria) and related it to age at training start. In this analysis, training duration showed no systematic dependence on age at training start (Figure R6), indicating that animals that started training at an older age did not consistently learn more slowly.

The age dependence we previously observed when using **age at recording** (Figure 1S1c,e) therefore seems to reflect that “age at recording” is a downstream variable that partly incorporates training duration. In other words, our previous analysis was misleading: it seems not to be the case that older animals (at training start) take longer to learn the task, but rather that those animals who take longer to learn the task are older by the time they reach the recording stage. Thank you for pointing out this confusion.

Second, to directly test whether training duration accounts for the reported age effects, we fitted extended models including both age and training duration for each metric (Table R2). **Crucially, the age effects were largely unchanged after controlling for training duration,** indicating that the main age-related conclusions are unlikely to be explained by slower learning or longer training. Training duration itself explained additional variance for a subset of metrics, but this did not remove or reverse the age effects. For several metrics (pre-stimulus firing rate, firing-rate contrast-modulation slope, FF quenching, and FF contrast-modulation slope), we found evidence for no additional effect of training duration (moderate/strong evidence for H0). In contrast, for a subset of metrics (post-stimulus firing rate and pre/post-stimulus FF), training duration showed evidence for an effect (moderate/strong evidence for H1).

This insight motivated two concrete revisions: (1) we toned down and corrected statements regarding age and learning speed; and (2) we added a brief section summarizing these control analyses in the revised manuscript (Results, p. 11-12, lines 24–4) and updated the relevant supplementary figure and Table S1,2 accordingly.

"**Age effects on neural variability persist after accounting for training duration**

Age-related effects measured at recording could, in principle, be partly entangled with differences in training history, as mice vary substantially in learning speed. Although individual training duration explains only a small fraction of variance in age at recording within our dataset (Figure 1-S2), and we did not observe slower learning in older mice (Figure 1-S1), training duration in the IBL task varies systematically across animals and laboratories (International Brain Laboratory et al., 2023). Thus, the neural differences we observe could reflect older animals having followed a longer training trajectory rather than chronological age per se. To address this alternative explanation, we quantified each animal's training duration and tested its association with our neural metrics.

To directly compare the two predictors, we fitted extended models including both age and training duration for each metric. Across metrics, the evidence for age effects was essentially unchanged after adding training duration (Table S1, S2): pre- and post-stimulus firing rates, post-stimulus Fano Factor, and FF quenching all retained strong evidence for an age effect in both the age-only and extended models.

Training duration showed limited, metric-specific contributions and did not change conclusions about effects of age (Table S2). Training duration provided strong additional evidence for post-stimulus firing rate and moderate additional evidence for pre-, post-stimulus Fano Factor, but did not reduce the evidence for age. For several metrics (e.g., pre-stimulus firing rate, firing-rate contrast-modulation slope, FF contrast-modulation), the extended models instead provided strong evidence against a training-duration effect. Critically, for Fano Factor quenching, the joint models continued to favour an age effect while providing moderate evidence for the absence of a training-duration effect, indicating that reduced neural variability quenching in older animals is not explained by differences in training duration."

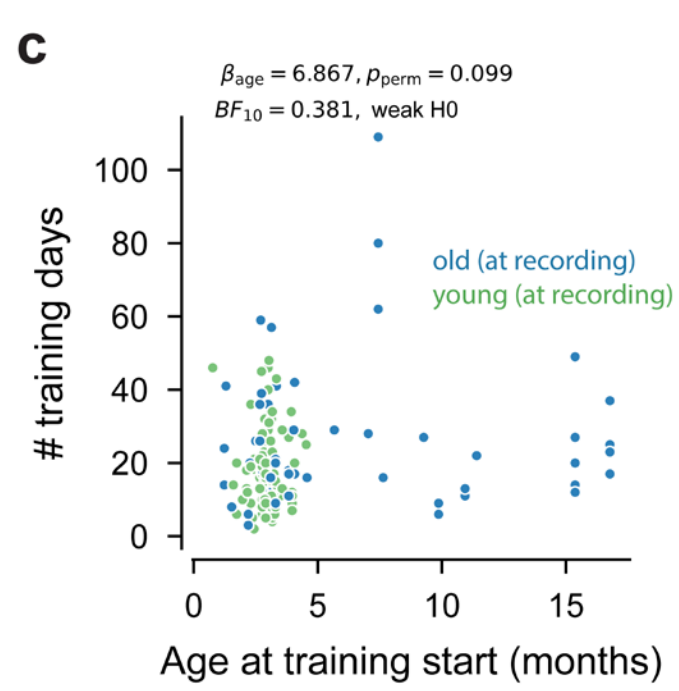

Figure R6 (Figure 1 S1c). Relationship between age at training start and training duration. Number of training days each mouse took to reach the behavioral criterion. Each point is one mouse. Colors represent the age groups (blue = old, green = young, groups are defined by age at recording, for consistency with the main figures). Conclusions are the same when we use a slightly different definition of training duration: number of days, sessions and trials required to reach the behavioural criterion or cumulative days, sessions and trials completed before the first neural recording.

metric	β_age	BF_evidence_age
rt_median	-0.014	<0.1, strong H0
rt_CV	+0.158	>100, strong H1
pre_fr	+0.216	>100, strong H1
post_fr	+0.229	>100, strong H1
fr_delta_modulation	+0.373	0.28, moderate H0
pre_ff	-0.001	<0.1, strong H0
post_ff	+0.056	>100, strong H1
ff_quench	+0.070	>100, strong H1
ff_quench_modulation	-0.066	1.42, weak H1

Table R1 (Table S1). Age effects from the original models (in the main analyses). For each of the key behavioral and neural metrics, the table reports β and BF_{10} for age.

metric	β_age	BF_evidence_age	β_training	BF_evidence_training
rt_median	-0.025	0.22, moderate H0	+0.483	0.49, weak H0
rt_CV	+0.140	>100, strong H1	+0.276	0.37, weak H0
pre_fr	+0.211	>100, strong H1	+0.311	<0.1, strong H0
post_fr	+0.202	>100, strong H1	+1.200	>100, strong H1
fr_delta_modulation	+0.394	0.42, weak H0	-0.946	<0.1, strong H0
pre_ff	-0.012	<0.1, strong H0	+0.449	7.81, moderate H1
post_ff	+0.045	13.6, strong H1	+0.424	8.87, moderate H1
ff_quench	+0.077	>100, strong H1	-0.297	0.25, moderate H0
ff_quench_modulation	-0.072	3.16, moderate H1	+0.310	<0.1, strong H0

Table R2 (Table S2). Models controlling for training duration: extended models with both age and training duration. For each behavioural and neural metric, we report results on an extended model including both age at recording and training duration. In the extended models, the age term reflects the partial age effect controlling for training duration; the training term reflects any additional contribution of training duration beyond age.

R1.3. The authors report that the main statistical method used in the paper is a generalized linear model. However, examining the methods section, it appears the authors actually used a general linear model. This terminology is commonly confused in the literature, so I think it's important to clarify. A general linear model is linear Y ~ WX with residual errors assumed to be gaussian distributed, whereas a generalized linear model is nonlinear and "generalizes" to non-gaussian noise models Y ~ f(WX). My apologies if I misunderstand the approach used. In most of the plots that use a regression, the y-axis is strictly positive, and in some plots the distribution appears to have a long tail towards large values. This makes me think that a general linear model with a gaussian noise model may be in-appropriate for this data. This issue seems the most pronounced in the insets of Fig 1C.

Please clarify the statistical method used - general linear model or generalized linear model. If a general linear model was used please comment on whether the residuals were approximately normally distributed or use an alternative approach to deal with non-normal data. If a generalized linear model was used, please report the link function and noise model used.

We thank the reviewer for pointing out the ambiguity in our terminology and for raising the issue of distributional assumptions.

We have now clarified in the Methods that the regressions reported in the manuscript use **general linear models** applied to continuous summary metrics. These models were implemented in a generalized linear modelling framework with a **Gaussian family and identity link**, which is equivalent to ordinary least-squares linear regression.

Because several of the outcome variables are strictly positive and right-skewed, we took additional steps to address the reviewer's concern. In the neural analyses, Fano factors and firing rates **were already analysed on a log scale**. Following the reviewer's suggestion, we now apply the same principle to strictly positive and right-skewed behavioural metrics: median RT and the psychometric threshold are analysed using log-transformed values. In contrast, RT variability, signed change indices (e.g. FF quench and modulation metrics), nonnegative metrics with substantial mass near zero (absolute bias), and bounded proportions (mean lapse) are analysed on their natural scale, since log transforms are undefined at zero and can introduce artificial heavy lower tails for distributions concentrated near zero.

We examined residual Q–Q plots for the most right-skewed behavioural metrics and for representative neural metrics under these transformations. For log-transformed median RT, psychometric threshold, and Fano factors, the residuals show approximate linearity with mild deviations in the tails, and no gross violations such as multimodality. For RT variability, analysed on its natural scale, residuals were close to linear with only mild tail deviations. For quench indices, residuals exhibit somewhat heavier tails, as expected for difference-type measures, but remain unimodal (example Q-Q plots in Figure R7).

Importantly, our **main statistical inferences do not rely solely on Gaussian residual assumptions.** For each metric, we complement the regression estimates with permutation tests (shuffling age labels) and Bayes Factors computed on the analysed summary metrics. Additionally, **analyses on the raw versus transformed scales yield qualitatively identical BF conclusions**, and the overall conclusions of the paper are unchanged after these additional checks and diagnostics (Table R3).

We have revised the Methods section to (1) use the term *general linear model* consistently for Gaussian identity-link regressions, and (2) describe the transformations and residual diagnostics used in the analyses (Methods, p. 22, lines 28–42).

> "To test for a statistically significant relationship between each metric and mouse age, we used the slope of age (β_{age}) from a general linear model (Gaussian family, identity link, using a generalized linear modelling framework) as the test statistic. Strictly positive

and right-skewed measures (median RT, psychometric threshold, firing rates, Fano factors) were analysed on a log scale to reduce skewness and stabilise variance. In contrast, RT variability, signed change indices (e.g. FF quench and contrast modulation metrics), nonnegative metrics with substantial mass near zero (absolute bias), and bounded proportions (mean lapse) were analysed on their natural scale, since log transforms are undefined at zero and can introduce artificial heavy lower tails when values are concentrated near zero. We inspected residual Q–Q plots for representative models; residuals for log-transformed metrics showed approximate normality with mild tail deviations, whereas difference-type indices and bounded/near-zero metrics exhibited somewhat heavier but unimodal tails. Importantly, we verified that our conclusions did not change depending on the log-scaling of these variables. All main inferences about age effects are based on Bayes Factors computed on these summary statistics and additional permutation tests with age-label shuffling."

metric	β_raw	BF_evidence_raw	β_log	BF_evidence_log	stable_conclusion
abs_bias	+2.990	26.1, strong H1	+1.347	12.2, strong H1	yes
threshold	+6.005	>100, strong H1	+0.348	>100, strong H1	yes
mean_lapse	+0.029	>100, strong H1	+1.452	23.5, strong H1	yes
rt_median	+0.002	<0.1, strong H0	-0.014	<0.1, strong H0	yes
rt_CV	+0.158	>100, strong H1	+0.230	>100, strong H1	yes

Table R3. Robustness of behavioural age-only regressions to outcome scaling. We compare results obtained on the original (raw) scale versus log-transformed values. Across all behavioural metrics, the log transform did not change the sign of the estimated age effect and did not alter the Bayes-factor conclusion category. Note, only median RT and the psychometric threshold are analysed using log-transformed values in the final analyses.

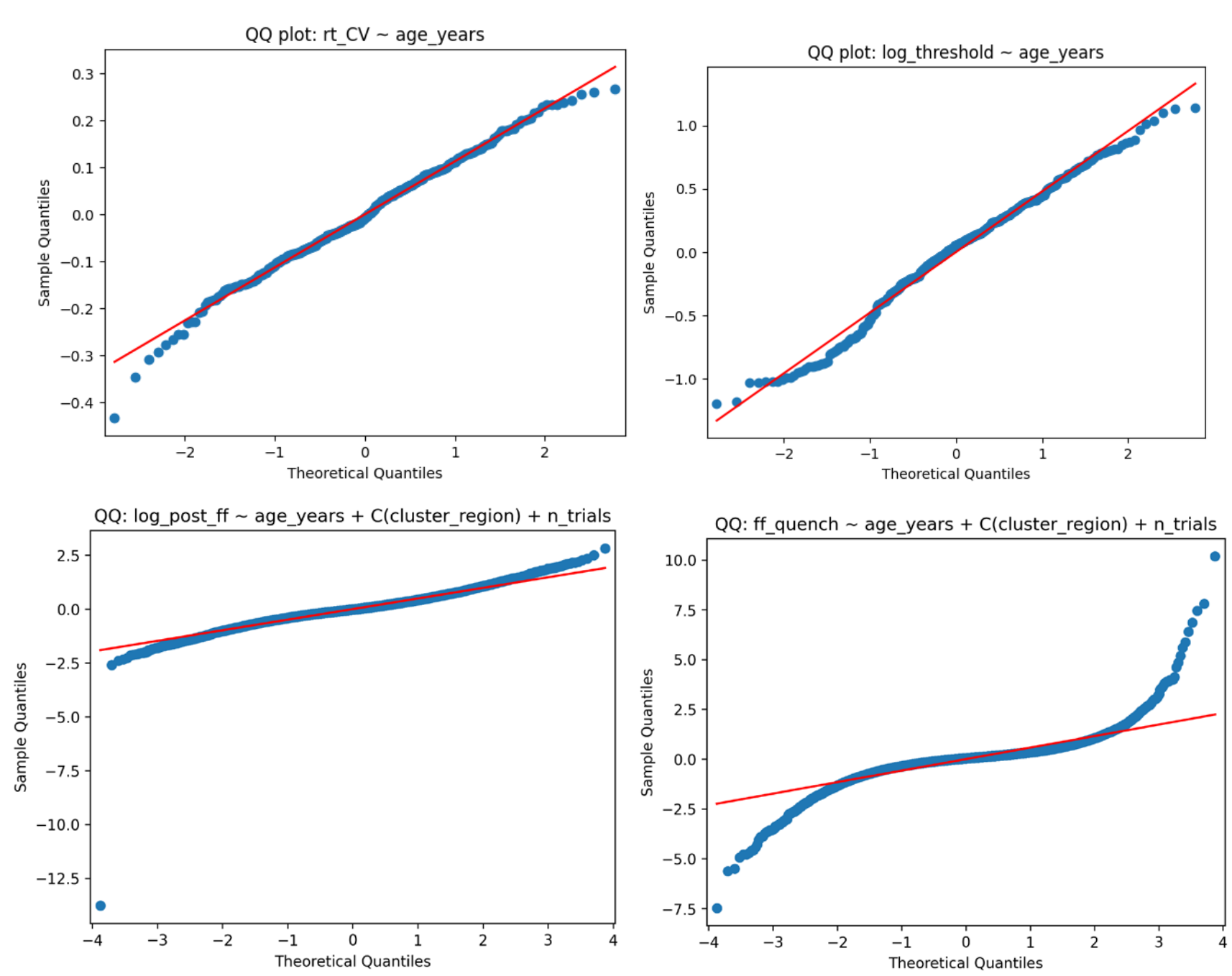

Figure R7. Residual Q–Q plots for representative behavioural and neural regression models. (a) Raw RT variability (CV) regressed on age. (b) Psychometric threshold σ on the log scale regressed on age. (c) Log-transformed post-stimulus Fano factor regressed on age, with region and trial-count covariates. (d) FF quench index regressed on age, illustrating heavier tails for difference-type metrics. Pearson residuals are shown with fitted reference lines (line = "s"). These diagnostics show approximate linearity for log-transformed positive right-skewed metrics, with mild tail deviations, and heavier but unimodal tails for bounded or difference-type indices.

R1.4. It is not clear to me whether the regression analyses in Figure 3 and 4 were computed on a cell by cell basis or a probe insertion by insertion basis. Was each of the neural metrics (firing rate, contrast modulation slope, FF, etc) computed for each cell, or averaged for each insertion? Computing these metrics on a cell by cell basis is the standard approach and most interpretable, but the plots in Figure 3/4 show the regression line over the data grouped by probe insertion. Further the methods section does not clarify the approach used, since neural yield must be computed on an insertion basis but neural yield is listed alongside other neural metrics. Please clarify how the regression analyses were computed. If analyses were computed on metrics averaged for each insertion, please justify this approach.

We thank the reviewer for pointing out that the analysis level was not sufficiently clear. Neural yield is inherently defined and analysed at the probe-insertion level. By contrast, all other neural metrics (firing rate, firing-rate contrast modulation slope, Fano Factor, Fano Factor quench, and Fano Factor contrast-modulation slope) were computed at the single-neuron level, and all statistical analyses, including the regression models underlying Figures 3 and 4, were performed on these neuron-level metrics. For visualization, however, Figures 3 and 4 show probe-insertion-level summaries (each point corresponds to the average of neurons recorded on one probe) to improve readability, while the overlaid regression lines show the predicted relationship from the neuron-level regression models.

We have clarified these distinctions in the Methods and figure captions. (Methods, p. 22, lines 17–26; Figure 3, Figure 2 S1 captions)

> "We used Bayes Factors and complementary p-values to test the effect of age on each behavioral and neural metric. Behavioral metrics (absolute bias, threshold, mean lapse rate, median RT, and RT variability) were computed at the session level. Neural metrics included neural yield, firing rate, contrast modulation of firing rate, Fano Factor, Fano Factor quench, and contrast modulation of Fano Factor. For each neuron, contrast-modulation slopes were obtained by fitting a linear regression to the change in firing rate (Δ firing rate, post- minus pre-stimulus window) or Fano Factor (Δ Fano Factor, post-minus pre-stimulus window) across all contrast levels. Neural yield was defined and analysed at the probe-insertion level, whereas all other neural metrics were computed at the single-neuron level, and all statistical analyses were performed on these neuron-level metrics."

Minor concerns

R1.5. It is not clear to me how to interpret mouse age against literature reports from other species - the authors cite studies in humans, monkeys, and cats. I would appreciate a brief review of how results in the literature have defined young/old groups in other species. I appreciate the citation of Cottam et al, 2025 which roughly maps mouse age 3-20 months to human 23-70, this helps provide meaningful context. Adding this reference in the introduction would help clarify the focus of this study - middle age of life and not adolescence or elderly populations.

We agree that clearer cross-species age context is important. Across species, ageing studies typically compare adult with late-adult or aged groups. In humans, “young” samples are usually in their 20s or 30s, whereas “older” samples are often above about 60 years, and in some studies extend up to ~85 years of age. The non-human primate and cat studies we cite similarly focus on late-adult/aged animals; for example, rhesus macaque studies compared young adults with very old animals (5–9 vs 23–31 years), with the aged group reported to correspond to ~70–90-year-old humans and the young-adult group with an approximate human-equivalent age of ~18–30 years (Yang et al., 2009; Wang et al., 2019), and the cat study contrasted young adults with old cats (1–3 vs 12 years; Hua et al., 2006). Consistent with cross-species age-mapping, our 3–20-month age range mainly samples adult and middle-aged mice, rather than adolescence or very late-life, and our findings should be interpreted in this context.

In the revised manuscript, we also added the age ranges of key cited studies at their first mention, to help readers interpret our mouse age range in cross-species context.

> (Introduction; p. 2, lines 2-3) “Human studies of healthy ageing, typically comparing young adults (20–30 years) with older adults (often up to ~85 years), present a complex and sometimes contradictory picture of age-related changes in neural variability. ”
>
> (Introduction; p. 2, lines 30-31) “In anaesthetized rhesus monkeys, comparing young adults (5–9 years; ~18–30 human-equivalent years) with aged animals (23–31 years; ~70–90 human-equivalent years), cortical areas V1 and MT show increased age-related trial-by-trial spiking variability,...”
>
> (Introduction; p. 3, lines 27-29) “We analyzed extracellular Neuropixels recordings from 149 mice (aged 3–20 months; roughly corresponding to 23–70-year-old humans; Cottam et al., 2025) across 16 brain regions,...”

R1.6. When did mouse behavior and neural recordings happen with respect to each mouses’ light cycle? Is it possible that differences in chronotype with age interact with the findings of this paper?

We thank the reviewer for raising this point. Across laboratories, mice were housed under either a normal or inverted 12:12 light–dark cycle; accordingly, depending on the laboratory, behavioural training and recordings occurred in either the light or the dark phase of the mouse’s

cycle. In our dataset, five labs used an inverted light cycle (Angelakilab, Wittenlab, Churchlandlab, Hausslerlab, Churchlandlab_UCLA) and five used a non-inverted cycle (Cortexlab, Danlab, Mainenlab, Zadorlab, Steinmetzlab); two labs (Hoferlab, Mrsicflogellab) used mixed regimes.

To evaluate whether light-cycle regime could confound the age effects reported in the manuscript, we stratified sessions by lab light-cycle regime (inverted vs non-inverted) and inspected age distributions. Age coverage differed across subsets: the inverted subset (N=143) included many older sessions, whereas the non-inverted subset (N=161) was concentrated at younger ages with relatively few older sessions (Figure R8). This is due to the ageing dataset being mostly collected in the Churchland lab at CSHL (see response to R1.1).

To test the reviewer's concern directly, we fitted joint models including light-cycle as a categorical covariate and an age×light-cycle interaction for both key behavioural measures (median RT and RT variability). Bayes Factors did not support a light-cycle main effect (median RT: BF_{10}=0.54, weak H0; RT variability: BF_{10}=0.14, moderate H0) nor an age×light-cycle interaction (median RT: BF_{10}=0.90, weak H0; RT variability: BF_{10}=0.65, weak H0). Together, these results suggest that the age-related increase in RT variability is unlikely to depend on light-cycle regime.

More broadly, light-cycle regime is tracked in IBL metadata and was evaluated as a potential source of between-lab variation in training outcomes; in that analysis, light cycle contributed little to predicting training duration compared to behavioral features (International Brain Laboratory et al., 2023). In line with this, we have wondered about the effects of the light cycle in our lab previously. An MSc student has analyzed the public IBL behavioral dataset to test whether learning speed differed between inverted vs non-inverted light cycles. The results were mixed and sensitive to the operational definition of learning speed and training consistency, and did not provide a robust, generalizable advantage of either light-cycle regime (Wulfse, 2023).

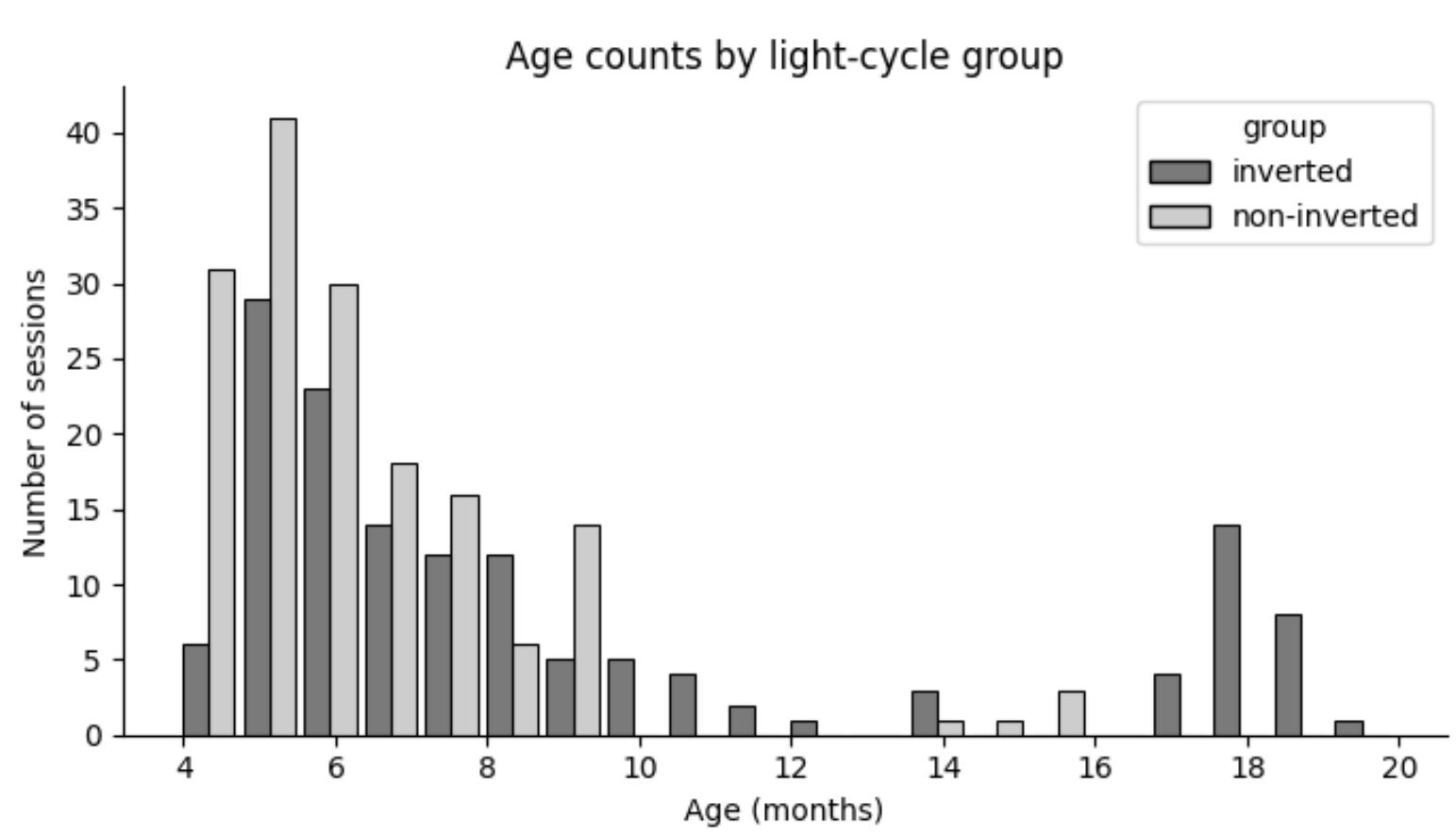

Figure R8. Age distribution by light-cycle group. Age distribution for laboratories using an inverted (dark grey) or non-inverted (light grey) light cycle. Inverted: N=143 sessions; non-inverted: N=161 sessions.

R1.7. On pg 4, "older animals do not strongly differ in the degree…(Figure 1-S3; Findling et al, 2023). Please clarify the results from this study versus those in the reference Findling et al 2023.

We thank the reviewer for raising this point. Findling et al. (2023) characterize history-dependent choice biases in the IBL task but do not examine how these effects vary with age. In our study, age was treated as a continuous variable, and we specifically tested whether block- or history-dependent shifts in psychometric parameters scale with age. As shown in Figure 1 S3, we did not observe age-related modulation of these history-dependent effects, indicating that the age-related changes we report in overall psychometric parameters are not explained by altered integration of prior information. We have clarified this distinction in the revised text.

> (Results; p. 4, lines 36-41) "We did not observe age-related changes in choice bias conditioned on blocks or previous choices (Figure 1-S4), suggesting that the age-related differences in overall psychometric parameters are not driven by changes in the integration of prior information with sensory evidence. While history-dependent biases are well established in this task (Findling et al., 2023), they do not seem related to the age effects observed here."

R1.8. On pg 4 "Older mice did not respond more slowly, but were more variable in their RTs". This implies that older mice sometimes have faster reaction times than younger mice. This appears to be true in Figure 1G, the older mice have more probability mass in the first three time bins. My understanding is that trials with RTs < 80ms were excluded. Were young mice more impulsive, and thus more heavily impacted by this 80ms cutoff?

We thank the reviewer for raising this point. Because it is closely related to Comment R1.10, we address it together there. Please see our response to R1.10.

R1.9. Does the regression on the inset of Fig 1D use all contrast levels? Would you get the same result if you only looked at contrast = 0 trials? I'm nitpicking here along with concern #8 because I'm interested in the possibility that older mice use cognitive effort in a more dynamic way - longer focus/RT on hard trials, and less on easier trials.

We thank the reviewer for this thoughtful comment. The regression shown in the inset of Figure 1d is based on median RT per session computed across all contrast levels. This choice reflects our aim to summarize overall RT changes with age across the full range of task difficulty.

To test whether our conclusion depends on pooling contrasts, we repeated the analysis using only trials in which no stimulus was present (contrast = 0). As shown in the new Figure R9, even when restricting the analysis to those trials, the data do not provide robust evidence that older mice have longer median RTs.

The reviewer's suggestion that older mice might deploy cognitive effort in a more dynamic, trial-type–dependent manner is intriguing. Current work in the lab is exploring these ideas by fitting

models of trial-to-trial engagement states to response times (Johnson et al., 2025), which we plan to extend to ageing.

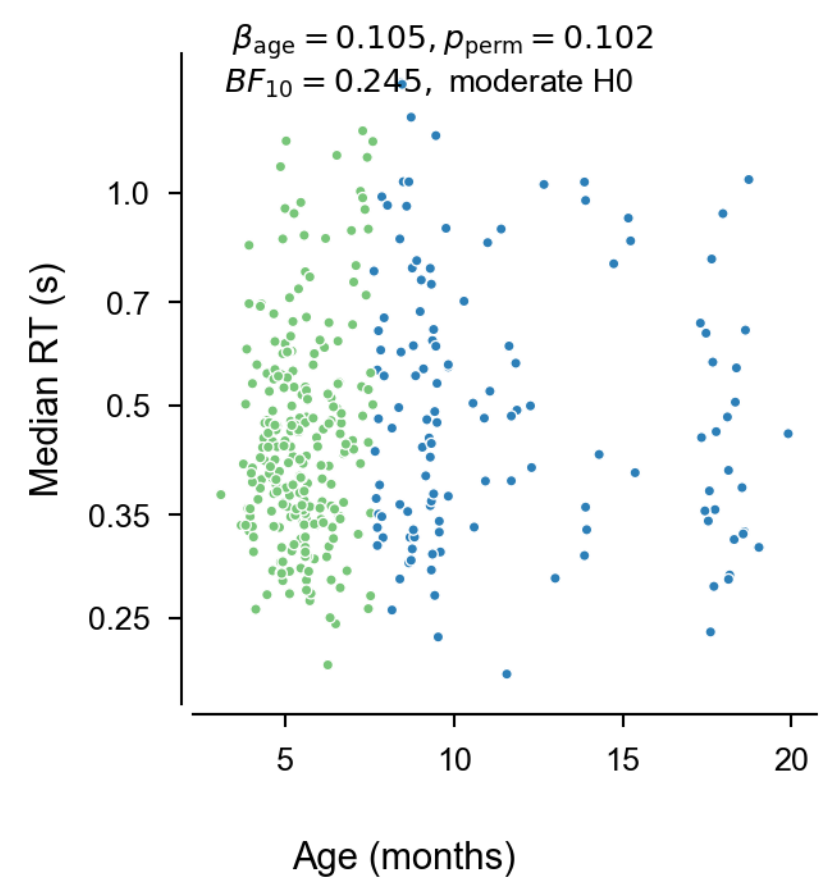

Figure R9. Median response times on zero-contrast trials as a function of age. Each dot represents one session. Note the log-scale on the y-axis.

R1.10. Was there any difference in premature responses, or other violation type trials/responses with age?

We quantified several "violation-type" trial categories in the first 400 trials of each session: (1) timeouts/no-go trials (choice = 0), (2) anticipatory responses with RT < 80 ms, (3) very slow responses with RT > 2 s, and (4) "overall excluded trials", defined as the union of (1)–(3) and matching the exclusion criteria used for our main RT analyses. For each category, we counted the number of trials per session and related this count to age using the same linear model, permutation test, and Bayes factor approach as in the main text (Figure R10).

Timeouts and very slow responses (RT > 2 s) showed no systematic age dependence (Figure R10a, c), indicating that older mice were not more prone to lapses or disengagement.

In contrast, the number of anticipatory responses (RT < 80 ms) showed a positive association with age (Figure R10b). As also noted in Comment R1.8, this means that the <80 ms exclusion criterion removes slightly more trials from older mice, not younger mice. Importantly, such trials were rare and excluded by the 80 ms-2 s RT cutoff, making them unlikely to explain the age-related increase in RT variability.

Consistent with this, the total number of excluded trials per session did not increase with age (Figure R10d). Thus, ageing does not lead to more invalid trials overall, and the age-related increase in RT variability is unlikely to be driven by differences in such trials.

We also updated the supplementary Figure 1 S2 in the manuscript with above analyses.

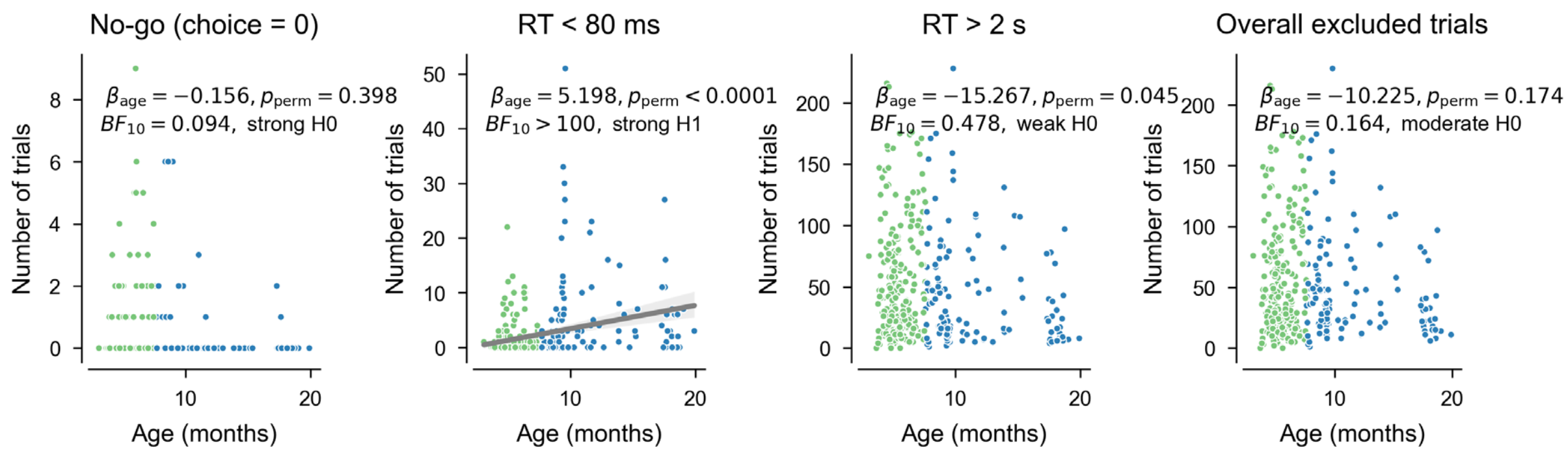

Figure R10 (included in Figure 1 S3). Relationship between age and the number of different "violation-type" trials in the first 400 trials of each session. (a) Timeouts/no-go trials (choice = 0). (b) Anticipatory responses with RT < 80 ms. (c) Very slow responses with RT > 2 s. (d) "Overall excluded trials", defined as the union of (a)–(c), corresponding to the RT-based exclusion used in the main analyses. Each dot represents one session; lines and shaded regions show the regression fits and their 95% confidence intervals.

R1.11. How was the cutoff of 2s for RT determined? It appears the young mice have a longer tail in Fig 1G, so this cutoff may influence the behavioral conclusions of Fig 1.

We used the same RT inclusion window (80 ms-2 s) as in previous analyses of this task within the International Brain Laboratory framework (International Brain Laboratory, Benson, et al., 2025). This range is intended to exclude very fast responses that are unlikely to be stimulus-driven and very slow responses that are typically interpreted as lapses or disengagement rather than meaningful decision-related variability. Importantly, we chose to adhere to the standard IBL QC conventions for both behavioral and neural analyses to facilitate direct comparability with existing and future studies using the same dataset and processing framework.

To address the concern that the 2 s cutoff might differentially affect age groups, we explicitly quantified the number of trials with RT > 2 s (as well as no-go trials and RT < 80 ms) in the first 400 trials of each session (Figure R10; see response to R1.10). Very slow responses did not show clear age dependence, and the overall number of excluded trials per session (union of no-go, RT < 80 ms, RT > 2 s) also did not increase with age. Thus the 2 s cutoff does not systematically remove more trials from older mice and therefore is unlikely to account for the age-related increase in RT variability reported in Figure 1.

We have clarified this in the revised text (Results; p. 4, lines 26-28):

> "Additionally, only trials with RTs between 80 ms and 2 s were included, to exclude anticipatory (<80 ms) and very slow (>2 s) responses that are unlikely to reflect stimulus-driven decisions, consistent with prior IBL analyses (International Brain Laboratory et al., 2024; Figure 1g)...The number of in- or excluded trials did not show a significant correlation with age (included trial counts: Figure 1-S3c; number of trials excluded by RT filtering: Figure 1-S3b)"

R1.12. Does the confidence interval in Fig 3A take into account the hierarchical nature of the neural data? My understanding is that when computing p-values all cells from each session were shuffled together, it's not clear if a similar approach was used in Figure 3A.

The shaded confidence bands in Figure 3a were computed by pooling neurons within each age group and therefore do not explicitly account for the hierarchical structure of the data (neurons nested within probes). This panel is intended as a descriptive visualization of the data, and we do not base any formal statistical conclusions on it.

We have clarified this in the revised caption of Figure 3 (p. 10, lines 3-5):

> "Shaded areas indicate 95% confidence intervals obtained by pooling neurons within each age group and are provided for visualization only. Statistical inferences about age effects are based on permutation tests and Bayes factors as described in the Methods. "

R1.13. Fig 3H, this panel appears to not be referenced anywhere in the text.

We thank the reviewer for catching this oversight. In the original submission, the Swanson maps were not explicitly referenced in the main text; we have now added references to these figures when describing the regional patterns for metrics with evidence for a global age effect.

Figure 3h shows the Swanson map of region-wise age coefficients for firing rate contrast modulation slope. For this metric, the omnibus analysis does not provide strong evidence for a global age effect (Figure 3g). Our general strategy is therefore to refrain from over-interpreting regional patterns when the global effect is not supported, and to present the Swanson map primarily for completeness. In the revised manuscript we now make this explicit and briefly reference Figure 3h in the main text, as well as clarify its role in the figure caption.

> (Results, p. 8, lines 1-3) "...Region-wise estimates are shown in the corresponding Swanson map (Figure 3h) for completeness, but we do not interpret these regional patterns further given the lack of a robust omnibus effect."

> (Figure 3 caption, p. 10, lines 15-17) "... Because the omnibus analysis does not support a global age effect, this Swanson map is shown for completeness and is not interpreted in detail in the main text."

R1.14. Fig 4A. It appears that the FF increases for older mice with stimulus onset, peaking around 100ms compared to around 0ms for younger mice. What is the source of this increase in variability?

We observed a brief increase in FF immediately following stimulus onset, which was more pronounced in older mice than in younger mice. This transient elevation was not specifically anticipated, as our primary expectation was a post-stimulus quenching of variability, which is clearly present in both age groups.

At present, we do not know the source of this short-lived increase. Importantly, it is temporally confined and does not affect the age-related differences quantified in our predefined pre- and post-stimulus windows. We therefore highlight it as an interesting observation and a potential avenue for future work.

We have now noted this transient in the revised manuscript (Results, p. 10, lines 46–48):

> "We also note a short-lived transient Fano Factor increase immediately after stimulus onset (more pronounced in older mice), the origin of which remains to be determined."

R1.15. Please add line numbers to the text in order to help with reviewing.
As suggested, we have added line numbers to the manuscript.

Thank you again for those insightful and constructive comments.

Point-by-point reply to reviewer #2

This study provides comprehensive and standardized evidence of how aging affects behavior and neural activity in mice. The authors carefully investigated how choices during the International Brain Laboratory (IBL) task were modulated by aging. They found that older mice showed reduced choice accuracy and more variable reaction times compared to younger mice. The authors then examined neuronal activity during the IBL task and found that aging increased firing rates and post-stimulus neural variability in various brain regions. In particular, the authors focused on the increase in neural variability caused by aging.

Although this study does not include many results to support its conclusions, the relationship among decision-making, neural activity, and aging was not well understood in previous studies. This work provides essential findings on how aging affects the neural circuits involved in decision-making. I mainly have three major comments regarding the validation of (1) neural activity, (2) behavior, and (3) conceptual advances from previous literature in rodents.

We thank Reviewer 2 for their constructive comments and for recognizing the contribution of our study. We take these concerns seriously and have strengthened the analyses and clarified the presentation in the revised manuscript, as detailed below.

Major comments

R2.1. As reported in the author's previous work (Musall et al., Nat. Neurosci., 2019), neural activity is modulated by movement. This study showed that aging increased the variability of reaction times and neural activity after visual inputs. Was the modulation of activity merely a consequence of behavioral variability, or was it an effect of aging? The IBL behavioral setup includes a video camera; therefore, the authors can quantify and separate the neural modulation due to behavioral variability from that due to aging.

We thank the reviewer for raising this important point. Movement can indeed modulate neural activity, and we therefore performed additional control analyses to test whether the observed age effects on firing rate and trial-to-trial variability persist after accounting for movement.

First, we leveraged IBL video recordings to derive movement measures from DeepLabCut (DLC) tracking of the right camera. After applying IBL extended QC for the right camera and DLC (International Brain Laboratory, Benson, et al., 2025; International Brain Laboratory, 2022b), right-camera DLC measures were available for n = 343 sessions (Figure R11). We focused on two time-resolved movement features from the right camera: right paw speed (task-relevant) and nose-tip speed (task-irrelevant). We first examined their time courses aligned to stimulus onset (Figure R11a,d). We then summarized each feature within the same pre- and post-stimulus windows used to define our neural metrics. Across both features, we did not observe consistent evidence for a systematic association with age in either window (Figure R11b–c,e–f).

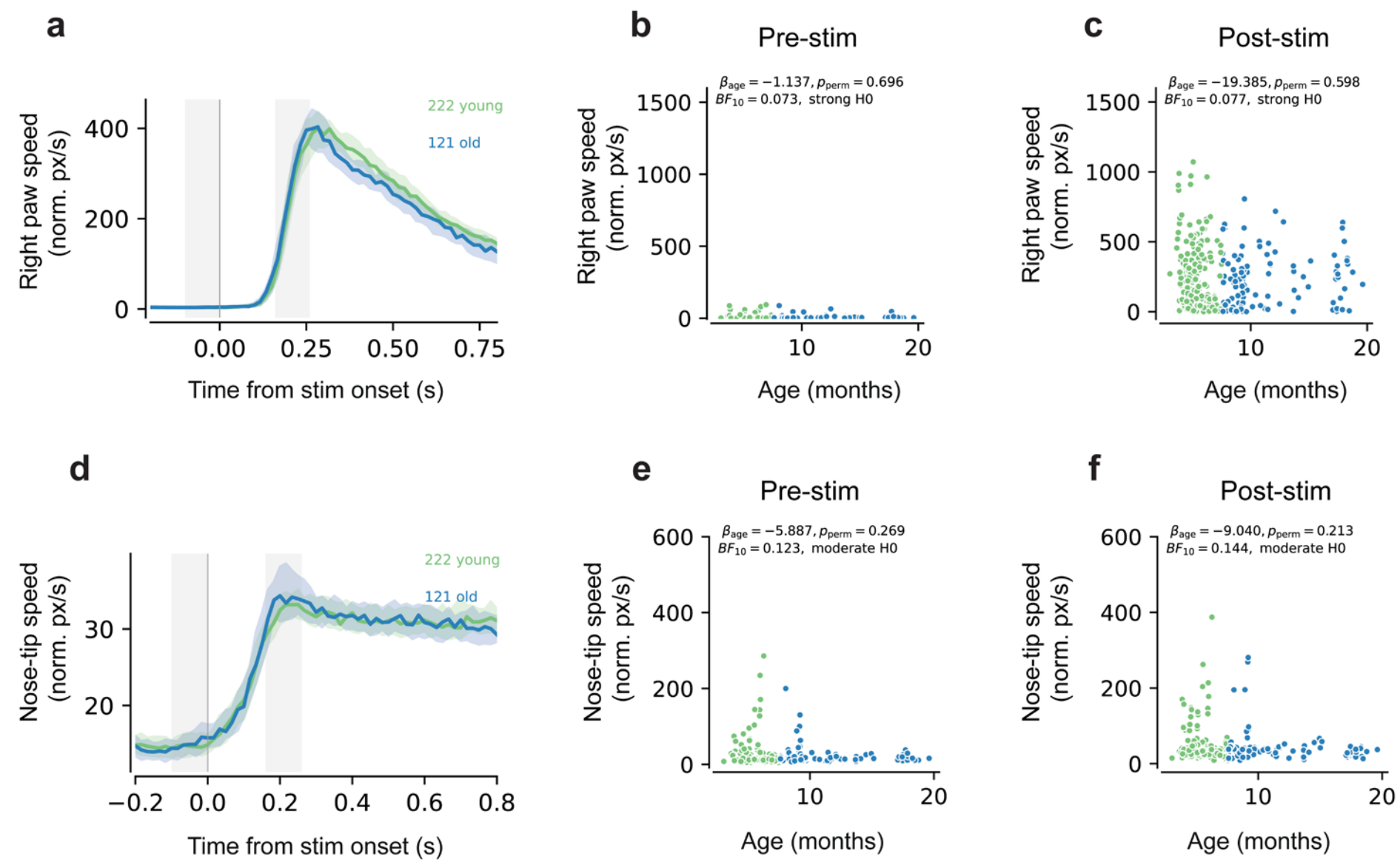

Figure R11 (Figure 4 S6). Right-camera DLC movement time courses and session-level summary metrics. (a) Right paw speed time course aligned to stimulus onset (t = 0), summarized across sessions (median ± 95% CI). Gray shaded regions indicate the pre-stimulus (−0.1 to 0 s) and post-stimulus (0.16 to 0.26 s) windows used to compute session-level movement summaries. **(b–c)** Session-wise median right paw speed within the pre-stimulus window (b) and post-stimulus window (c), plotted against mouse age; each dot corresponds to one session. **(d)** Nose-tip speed time course aligned to stimulus onset, summarized across sessions (median ± 95% CI), with the same pre/post windows indicated. **(e–f)** Session-wise median nose-tip speed within the pre-stimulus (e) and post-stimulus (f) windows versus age; each dot corresponds to one session.

Second, for each neural metric, we added both session-level movement covariates (right paw and nose-tip speeds) and age into the extended models. Movement covariates provided additional explanatory power in several models, but the main age-related conclusions were broadly preserved after adding these movement covariates (Table R4).

Only two metrics with initially weak-to-moderate evidence were sensitive to the inclusion of movement covariates: firing-rate contrast modulation shifted from moderate evidence for H0 to weak evidence for H1, whereas Fano Factor contrast modulation strengthened in favor of H1 (weak → moderate). All other metrics retained the same qualitative conclusion.

Together, these analyses indicate that the reported age-related differences in firing rate and variability metrics persist after accounting for the two video-derived movement readouts and are unlikely to be accounted for by these video-derived movement readouts alone.

metric	β_age	BF_evidence_age	β_paw	BF_evidence_paw	β_nose	BF_evidence_nose
pre_fr	+0.201	>100, strong H1	+0.016	0.37, weak H0	-0.028	>100, strong H1
post_fr	+0.211	>100, strong H1	+0.006	<0.1, strong H0	-0.027	>100, strong H1
fr_delta_modulation	+0.437	1.12, weak H1	-0.086	<0.1, strong H0	-0.119	3.7, moderate H1
pre_ff	+0.017	<0.1, strong H0	-0.015	6.14, moderate H1	+0.006	0.11, moderate H0
post_ff	+0.062	>100, strong H1	-0.014	7.58, moderate H1	-0.003	<0.1, strong H0
ff_quench	+0.052	11.7, strong H1	-0.002	<0.1, strong H0	-0.005	>100, strong H1
ff_quench_modulation	-0.076	5.81, moderate H1	-0.012	<0.1, strong H0	+0.014	0.45, weak H0

Table R4 (Table S3). Additional controls using video-based movement (right camera): Extended model summary (Age + Paw + Nose-tip). In sessions with QC-passing right-camera recordings (n = 343), we extracted session-level right-paw speed (task-relevant) and nose-tip speed (less task-specific) in pre/post windows and added them as covariates in the extended models. The table reports β and BF_{10} for age, paw, and nose-tip terms.

In the revised manuscript, we now include a brief section discussing movement and other potential contributors to the observed age effects (Results, p. 12, lines 7–25).

> "**Age effects are robust to video-based movement covariates**
> Neural activity is modulated by movement (Musall et al., 2019), including in well-trained animals (Yin et al., 2025), motivating a control analysis to test whether the reported age-related neural differences could be explained by movement. Using IBL right-camera videos, we extracted time-resolved paw speed (task-relevant) and nose-tip speed (less task-specific) (Figure 4-S6 a,d) from DeepLabCut tracking. Across QC-filtered sessions with valid right-camera tracking (n = 343), neither movement measure showed a systematic association with age in the pre- and post-stimulus windows used to define our neural metrics (Figure 4-S6, b-c,e-f). We next incorporated both movement measures (paw speed and nose-tip speed) as covariates in extended models for each neural metric (Table S3). Movement covariates provided additional explanatory power in several models, but the qualitative evidence for age effects was broadly preserved after including them. Only two metrics with initially weak-to-moderate evidence were sensitive to the inclusion of movement covariates (firing rate contrast modulation: moderate H0 → weak H1; Fano Factor contrast modulation: weak H1 → moderate H1). All other metrics retained the same qualitative conclusion.
>
> Together, these analyses suggest that while movement explains additional variance in firing-rate and variability metrics, the reported age-related differences persist after accounting for the two video-derived movement features and are therefore unlikely to be explained by these movement readouts alone."

We also explained the process in the Methods (p. 24, lines 15-31):

> "**Extended models including age and movement**

We used IBL right-camera videos to derive movement covariates from DeepLabCut tracking of two representative keypoints: right paw (task-relevant) and nose tip (task-irrelevant) (Figure 4-S6a, d). From the tracked x/y coordinates, we computed frame-to-frame speed (coordinates normalized by camera resolution and scaled by the camera sampling rate) and aligned the resulting speed time courses to stimulus onset. We summarized movement at the session level by computing median speed within the same pre- and post-stimulus windows used for neural metrics (pre: −0.1 to 0 s; post: 0.16 to 0.26 s), yielding four covariates (pre/post paw speed; pre/post nose-tip speed).

Sessions were required to pass IBL extended QC for the right camera (videoRight and dlcRight). This yielded n = 343 sessions with valid right-camera movement measures. We then fitted extended models for each neural metric by adding the movement covariates to the age-only models. Movement covariates were time-window matched: metrics defined in a single window used the corresponding pre- or post-stimulus movement measure, whereas metrics spanning both windows (e.g., Fano Factor quenching) included both pre- and post-stimulus movement covariates. All other modeling choices (predictors, covariates, log-transform conventions, and inference procedures) were identical to those described above."

Our movement controls were based on right-camera DeepLabCut tracking of two keypoints (nose tip and right paw), summarized as session-level speeds within task-relevant time windows. While these readouts capture salient, time-locked movements and improved model fit for several neural metrics, they do not provide an exhaustive characterization of whole-body movement. In particular, age-related differences may manifest in other movement components (or in more distributed motion energy) not captured by these two keypoints. We therefore added a brief note in the Discussion highlighting this limitation and suggesting that future work could extend these controls using richer video-derived measures:

(Discussion, p. 16, lines 7-10) "While we controlled for video-derived movement covariates (paw, nose-tip), future work could further refine such integrated predictors using richer behavioural characterizations (see Musall, Urai, et al., 2019), for example motion-energy–based video features or higher-dimensional, data-driven representations of behavioural modules (Wiltschko et al., 2015, 2020)."

R2.2. In the previous IBL study (Figure 2, International Brain Laboratory et al., eLife, 2021), the learning rates of mice depended on the laboratory. In this study, the newly added data on older mice comprise the main results. It is important to determine whether the aging effects observed in choice behavior during the electrophysiological and learning phases were not the result of laboratory differences. It is also important to analyze whether the behavioral differences reported here did not depend on when the data were collected, as the older-mice data were relatively recent.

We thank the reviewer for raising these important concerns. These points partly overlap with those addressed in R1.1. For completeness, we summarize the key control analyses and

reproduce the relevant figures here. In addition, we assess potential time-of-collection confounds. Overall, our results indicate that the age-related effects reported in this study are unlikely to reflect laboratory differences or time-of-collection confounds.

First, age is not confounded with laboratory or dataset. As shown in Figure R1 (copied below), mice of different ages are interleaved across labs, and Churchland-lab mice span a broad age range in both the original “brain-wide map” and the new “lifespan” datasets. Most of the ‘lifespan’ added dataset was recorded at CSHL in the late summer of 2020, when Covid-19 restrictions were briefly lifted in New York and allowed experiments to take place. In contrast, the “brain-wide map” dataset was collected from different laboratories over a longer period of several years.

To directly test robustness to dataset and laboratory differences, we repeated the main behavioural analyses when restricting the data to (1) the original IBL dataset only (Figure R2) and (2) sessions from the Churchland lab only (Figure R3). In both cases, the main age effects persisted (with reduced Bayes Factor evidence in the smaller subsets), arguing against an explanation in terms of lab- or dataset-specific differences.

Second, to assess potential time-of-collection confounds, we added a new diagnostic figure. Figure R12 shows that recording sessions from younger and older mice are distributed across overlapping recording periods and laboratories, indicating that mouse age does not map onto specific years or recording cohorts. This makes it unlikely that our findings are driven by when the data were collected.

Finally, all data were collected using standardised IBL behavioural and electrophysiological protocols, which further limits systematic differences between laboratories. Together, the continuous age distribution, cross-laboratory mixing of animals, and subset analyses provide converging evidence that the observed age-related behavioural effects reflect continuous variation with age rather than lab-, dataset-, or time-of-collection confounds.

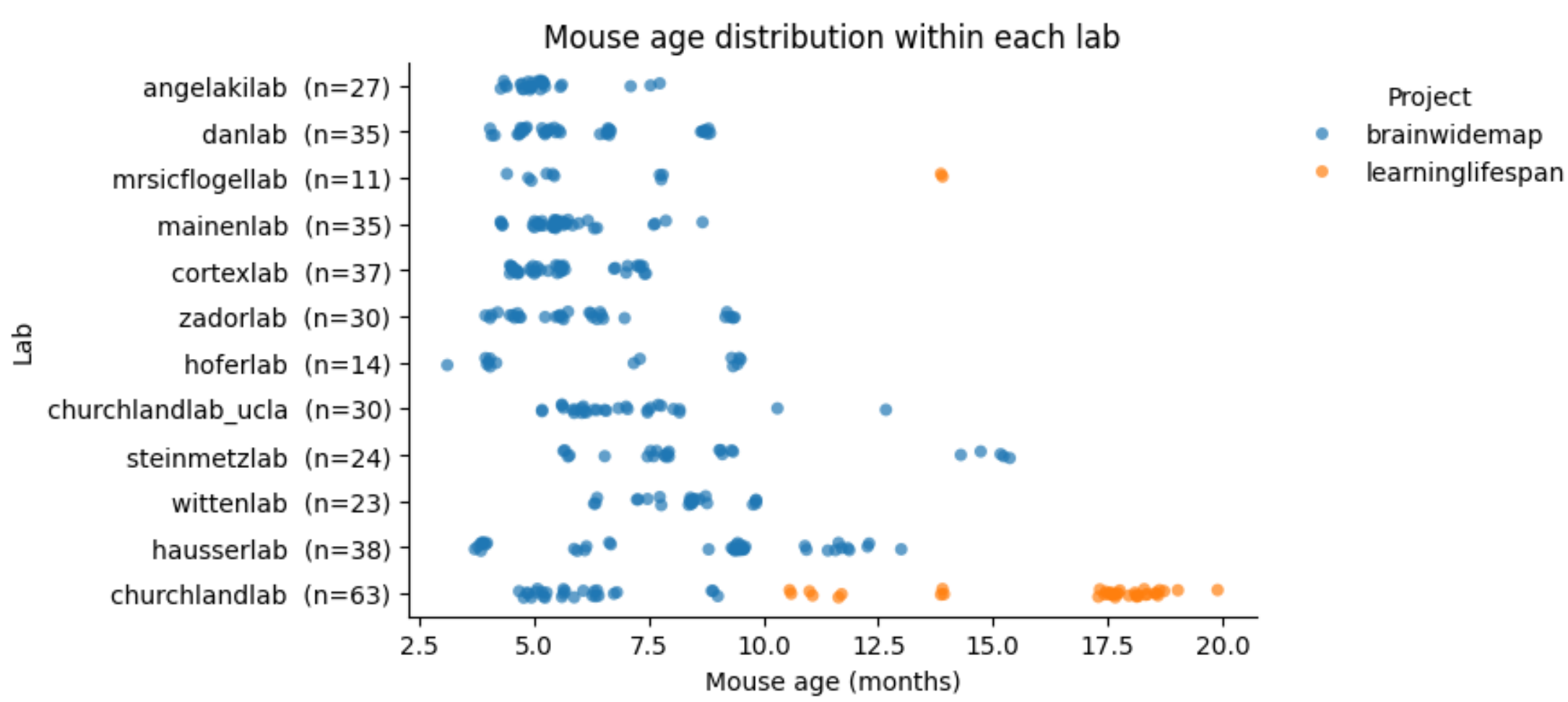

Figure R1. Distribution of mouse age across laboratories and datasets.

Each point represents one mouse, plotted by age at recording and grouped by laboratory. Colors indicate dataset (original IBL dataset “brain-wide map” vs. new dataset “lifespan”). Mice of different ages are interleaved across labs, and Churchland-lab mice span a broad age range in both datasets.

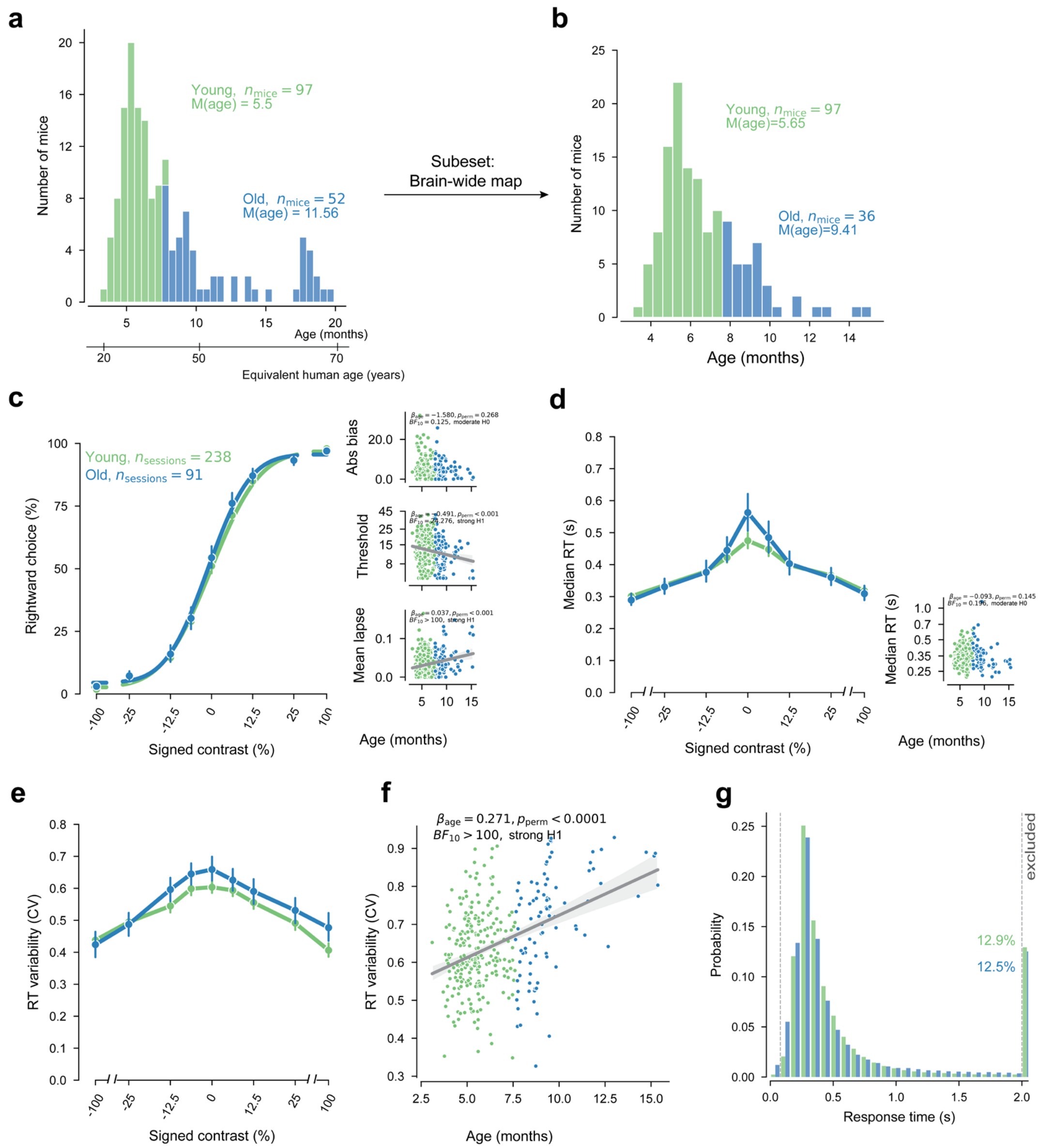

Figure R2 (Figure 1 S6). Replication of Figure 1 using only mice from the original IBL “brain-wide map” dataset. Age-related effects on RT variability is similar in direction and magnitude to those obtained in the full dataset.

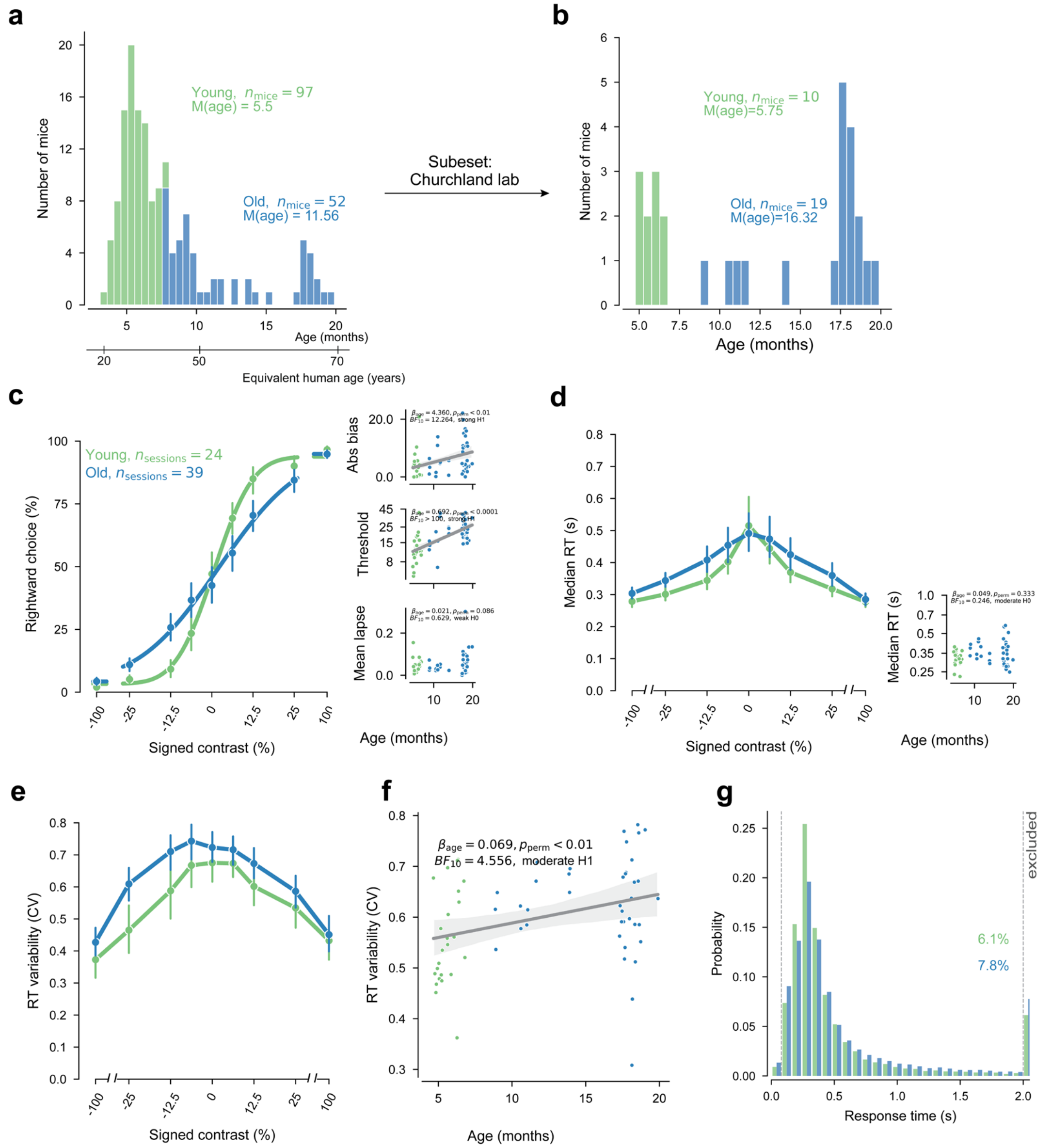

Figure R3 (Figure 1 S7). Replication of Figure 1 using only mice recorded in the Churchland lab.

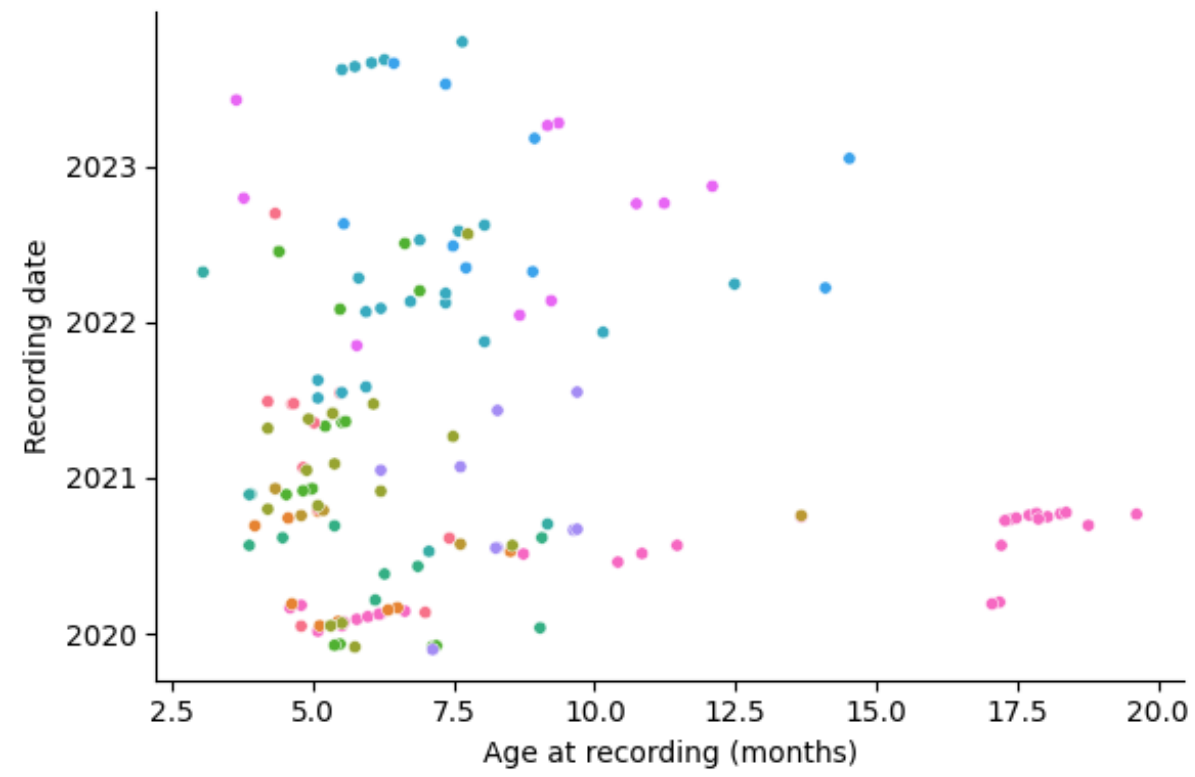

Figure R12. Distribution of mouse age and recording date across labs. Each point represents a recording session, plotted by the mouse's age at recording (x-axis) and recording date (y-axis). Colors indicate different labs. Recordings from older and younger mice are distributed across overlapping recording periods and labs.

R2.3. This study does not sufficiently cite previous rodent studies on aging. It has already been reported that aging modulates neural activity in rats and mice (Maziar et al., Cereb Cortex, 2023; Simkin et al., J Neurosci, 2015; Fischer et al., Cell Mol Life Sci, 2021; Randall et al., Neurobiol Aging, 2012). Effects of aging have also been reported in the field of Alzheimer's disease (Anastacio et al., Transl Psychiatry, 2022; Sosulina et al., J Neurochem, 2021; Wang et al., Behav Brain Res, 2017). Please summarize rodent studies on aging in the Introduction and Discussion to clarify the conceptual advances of the present study.

We thank the reviewer for highlighting the importance of the broader rodent ageing literature. In the revised manuscript, we have added a new paragraph to the Introduction that explicitly situates our work within prior rodent studies examining age-related changes in neural activity across multiple levels of analysis, including cellular, circuit, and disease-model approaches. We summarize this literature at a conceptual level to clarify how the present study complements previous rodent work.

> (Introduction, p. 2-3, lines 45–9) "Beyond primates, rodent studies have also reported age-related changes in neural activity across multiple levels of organization, from cellular and synaptic changes in excitability, synaptic transmission, and excitation–inhibition balance to circuit- and population-level alterations in firing rates and population activity patterns across regions (e.g., Randall et al., 2012; Simkin et al., 2015; Fischer et al., 2021; Turner et al., 2005; Hughes et al., 2010; Maziar et al., 2023; Barnes et al., 1997; Rosenzweig & Barnes, 2003; Barnes, 2011). Related changes have also been reported in rodent models of neurodegenerative disease, including Alzheimer's disease, which are often characterized by circuit hyperexcitability (H.-L. Wang et al., 2017; Sosulina et al., 2021; Anastacio et al., 2022). These studies span diverse experimental contexts, but often focus on one or a small number of regions and include many experiments conducted in passive paradigms or in anesthetized/ex vivo preparations. As a result, they provide limited leverage on how ageing impacts trial-to-trial neural variability during perceptual decision-making across brain regions."

Minor comments

R2.4. Page 4, paragraph 1 (Figure 1c). Older mice showed slightly worse performance both on the day they met this criterion and during their first Neuropixels recording session (Figure 1-S1d,f).
I was not sure how the behaviors between young and old mice were different on the day they met the training criteria. As far as I understand, the criteria were the same for all mice, so there should be no behavioral difference in the criteria (e.g. performance on easy trials).
Did the choice differences between young and old mice arise from (1) performance within the criteria, (2) performance outside the criteria, or (3) differences in how the criteria was achieved?

We thank the reviewer for raising this point. We would like to clarify that the training criterion was indeed identical for all mice: accuracy on easy trials exceeding 90% within a session. Thus, all mice met the same minimum performance threshold before entering the electrophysiological recordings.

However, meeting the minimum criterion does not imply identical behavioral performance. Even within sessions that satisfied the criterion, mice could differ in the exact level of performance above threshold (e.g., 91% vs. 98%). The observed differences between young and old mice, therefore, reflect variability within the criterion, rather than from differences in the criterion itself or in how it was achieved.

We also clarified this in the caption of Figure 1 S1 (p. 38):

> "(d) Performance on easy trials on the day each mouse reached criterion. The training criterion was identical for all mice. Observed differences reflect performance above this threshold within criterion-satisfying sessions on that day."

R2.5. Page 4, paragraph 4.
To validate variability, how did the authors decide to use different measurements for behavior (coefficient of variation, CV) and for neural activity (Fano factor)?

We thank the reviewer for this question. We used the coefficient of variation (CV) for behavioral variability and the Fano factor (FF) for neural variability. These choices were guided by a common principle aimed at capturing relative trial-to-trial variability through scale-normalized measures, while respecting the distinct statistical properties of behavioral and neural data.

Single-neuron spiking activity is commonly modelled as a Poisson-like process, in which the variance scales with the mean firing rate. The Fano factor estimates this mean–variance coupling and is therefore the standard measure of neural variability in the literature. In particular, we used a mean-subtracted Fano factor (Churchland et al., 2011) to avoid confounding variability estimates with condition-specific differences in mean firing rates when pooling trials across conditions. In contrast, response time variability does not follow Poisson statistics, and the CV provides an appropriate normalized measure by relating the standard deviation of response times to their mean.

Both measures are widely used in their respective behavioral and neural literatures, allowing our results to be directly compared with prior studies; in particular, several key studies in this line of work report single-neuron variability using the Fano factor (e.g., Yang et al., 2009; Wang et al., 2019; Liu et al., 2025). We also added a sentence to the Results to make this rationale explicit (p. 10, lines 29–31).

> "Reporting neural variability as Fano factor also facilitates direct comparison with prior work in this line of research (e.g., Yang et al., 2009; X. Wang et al., 2019; Liu et al., 2025)."

Importantly, our conclusions were robust to alternative measures of behavioral variability, like median absolute deviation (MAD) and standard deviation (SD) of log-transformed RTs (Figure 1-S5a).

Finally, we now explicitly mention in the Discussion that future work could complement FF with additional variability metrics to further refine the robustness and specificity of these effects (Discussion, p. 15, lines 14–17):

> "Future work could also examine complementary measures of neural variability beyond Fano factor to test the robustness and specificity of these effects (e.g., spike train irregularity; Holt et al., 1996; Nawrot et al., 2008)."

R2.6. Page 6, paragraph 1. Overall neural yield was slightly reduced in older mice.
It is interesting to observe that neural yield depends on age. It would be beneficial to provide a hypothesis to explain this observation.

We thank the reviewer for this comment. The observed age-related reduction in neural yield likely reflects a combination of technical (recording-related) and biological factors that broadly accompany aging. From a technical perspective, increased rigidity of the dura and greater tissue fragility in older mice may lead to increased superficial damage during probe insertion, reducing the number of well-isolated units. However, because the yield decrease was region-specific (most evident in PPC, but not in all cortical regions), insertion-related superficial damage alone is unlikely to fully explain the pattern.

In addition, aging is associated with neuronal loss and/or structural and cytoarchitectural changes across multiple cortical and subcortical regions, including alterations in neuronal density, dendritic structure, and synaptic integrity. Even in the absence of pronounced neuron loss, such changes can substantially affect neuronal health, firing reliability, and isolatability in large-scale extracellular recordings. These age-related biological factors may contribute to the reduced neural yield observed in older animals.

We have added a discussion of these potential explanations to the manuscript (Discussion, p.14-15, lines 44-7):

> "The slight reduction in overall neural yield observed in older mice may reflect a combination of technical (recording-related) and biological factors. From a technical

perspective, increased tissue and dural rigidity during probe insertion may increase superficial damage and reduce the number of well-isolated units; however, the regional specificity of the effect suggests that insertion-related factors alone are unlikely to account for the observed pattern. Biologically, ageing has been associated with modest and region-specific neuronal loss in some cortical and subcortical regions (Sabel & Stein, 1981; Yates et al., 2008; Stranahan et al., 2012). More broadly, ageing is accompanied by cytoarchitectural and structural remodeling, including changes in cellular organization, dendritic morphology, and synaptic integrity (Markham & Juraska, 2002; Morrison & Baxter, 2012). Such age-related changes may reduce the number of stable, well-isolated units obtained in large-scale extracellular recordings, even in the absence of pronounced neuron loss."

R2.7. Page 7, paragraph 1. Ageing increased firing rates globally.
Related to major comment 3: please include that increased firing rates have also been observed in rodents.

We have revised the manuscript to explicitly note that age-related increases in firing rates have also been reported in rodents. This clarification places our findings in the context of both rodent and primate literature.

(Results, p. 7, lines 17-26) "Ageing increased firing rates globally. Previous studies have reported increased firing rates with ageing across species. In rodents, age-related increases in firing rates have been reported in multiple brain areas, including sensory cortex and hippocampus **(**Hughes et al., 2010; Wilson et al., 2005; Bishop et al., 2022; see also Recanzone, 2018**).** Increased firing rates have also been reported in sensory cortex of other mammals, including cat V1 (Hua et al., 2006). In non-human primates, ageing has been associated with increased firing rates in cortical areas such as macaque V1 and MT (Schmolesky et al., 2000; Yang et al., 2009), as well as in specific cell types in the prefrontal cortex (Chang et al., 2005 in monkey PFC pyramidal cells). In contrast, other studies have reported decreased firing rates with ageing, particularly in prefrontal cortex (Wang et al., 2011 in monkey PFC)."

R2.8. I might be mistaken, but the range of color bar β(age) in Figure 3h appears larger compared to other figures. Does this imply that some brain regions (e.g. visual cortex) showed reduced contrast modulation, while other regions (e.g., hippocampus) showed increased modulation with aging? If so, please revise the last paragraph of page 7.

We thank the reviewer for this observation. Indeed, the range of β_{age} values in Figure 3h is broader than in other figures, reflecting heterogeneous and bidirectional regional trends in age-related contrast modulation rather than a uniform global effect. As shown in Figure 3h and in more detail in Figure 3 S4, contrast modulation slopes tend to decrease with age in some regions (e.g., visual cortex VISp+pm) and increase in others (e.g., hippocampal DG), which leads to no significant omnibus age effect across the brain (Figure 3g).

Consistent with our general interpretive framework throughout the manuscript, we describe regional patterns in detail only when a corresponding global age effect is supported. In this case, Bayesian factor analyses provided evidence for age-related effects only in a limited subset of regions (MOs and DG). We therefore refrained from emphasizing regional differences in the main text in order to avoid over-interpretation of effects that are not supported at the global level, while Figure 3h is intended to visualize regional differences in the direction and magnitude of β_{age}.

> (Results, p. 8, lines 3-6) “Notably, while β_{age} estimates showed heterogeneous and bidirectional regional trends (Figure 3h; Figure 3-S4), we describe regional patterns in detail only when a corresponding global age effect is supported, in order to avoid over-interpretation of region-specific effects.”

R2.9. Page 10, last paragraph. By contrast, for Fano Factor quenching we found strong evidence for no effect of training time,
(1) In the Methods section (Page 19), please add how the authors analyzed βtraining.
(2) This result implies that the training time and mouse age were different, although they were correlated. To clarify this paragraph, it would be worth developing a GLM that includes both βtraining and βage.

We thank the reviewer for the helpful suggestions. Following this suggestion, we performed additional control analyses to explicitly disentangle the effects of chronological age and training duration. Rather than examining training duration in isolation, we fitted extended models that included both age and training duration as predictors for each neural metric (Table R2, copied below; see also in our response to R1.2b). Crucially, the age effects were largely unchanged after controlling for training duration, indicating that the main age-related conclusions are unlikely to be explained by slower learning or longer training. Training duration itself explained additional variance for a subset of metrics, but this did not remove or reverse the age effects. For several metrics (pre-stimulus firing rate, firing-rate contrast-modulation slope, FF quenching, and FF contrast-modulation slope), we found evidence for no additional effect of training duration (moderate/strong evidence for H0). In contrast, for a subset of metrics (post-stimulus firing rate and pre/post-stimulus FF), training duration showed evidence for an effect (moderate/strong evidence for H1).

In addition to adding a brief section summarizing the analysis (Results, p. 11-12, lines 24-4), we have also clarified in the Methods how age and training duration were combined in extended models (Methods, p. 24, lines 1-13):

> (Results, p. 11-12, lines 24-4) “**Age effects on neural variability persist after accounting for training duration**
> Age-related effects measured at recording could, in principle, be partly entangled with differences in training history, as mice vary substantially in learning speed. Although individual training duration explains only a small fraction of variance in age at recording within our dataset (Figure 1-S2), and we did not observe slower learning in older mice (Figure 1-S1), training duration in the IBL task varies systematically across animals and

laboratories (International Brain Laboratory et al., 2023). Thus, the neural differences we observe could reflect older animals having followed a longer training trajectory rather than chronological age per se. To address this alternative explanation, we quantified each animal's training duration and tested its association with our neural metrics.

To directly compare the two predictors, we fitted extended models including both age and training duration for each metric. Across metrics, the evidence for age effects was essentially unchanged after adding training duration (Table S1, S2): pre- and post-stimulus firing rates, post-stimulus Fano Factor, and FF quenching all retained strong evidence for an age effect in both the age-only and extended models.

Training duration showed limited, metric-specific contributions and did not change conclusions about effects of age (Table S2). Training duration provided strong additional evidence for post-stimulus firing rate and moderate additional evidence for pre-, post-stimulus Fano Factor, but did not reduce the evidence for age. For several metrics (e.g., pre-stimulus firing rate, firing-rate contrast-modulation slope, FF contrast-modulation), the extended models instead provided strong evidence against a training-duration effect. Critically, for Fano Factor quenching, the joint models continued to favour an age effect while providing moderate evidence for the absence of a training-duration effect, indicating that reduced neural variability quenching in older animals is not explained by differences in training duration."

(Methods, p. 24, lines 1-13) "**Extended models including age and training duration**
To assess whether our age effects could be explained by training duration, we fitted extended models for key metrics that included both age and training duration as predictors simultaneously. Training duration was defined as the number of training days until the mouse first passed the standardised behavioural criterion and was expressed in years to match the age scale used in the model. All other modelling choices (predictors, covariates, and log-transform conventions) were identical to those described above.

In each extended model, we quantified evidence for including each predictor (age_years, training_duration) using Bayes Factors that compared otherwise identical models with and without the respective term. This allowed us to test whether age effects persisted after accounting for training duration, and whether training duration explained additional variance beyond age."

metric	β_age	BF_evidence_age	β_training	BF_evidence_training
rt_median	-0.025	0.22, moderate H0	+0.483	0.49, weak H0
rt_CV	+0.140	>100, strong H1	+0.276	0.37, weak H0
pre_fr	+0.211	>100, strong H1	+0.311	<0.1, strong H0
post_fr	+0.202	>100, strong H1	+1.200	>100, strong H1
fr_delta_modulation	+0.394	0.42, weak H0	-0.946	<0.1, strong H0
pre_ff	-0.012	<0.1, strong H0	+0.449	7.81, moderate H1
post_ff	+0.045	13.6, strong H1	+0.424	8.87, moderate H1
ff_quench	+0.077	>100, strong H1	-0.297	0.25, moderate H0
ff_quench_modulation	-0.072	3.16, moderate H1	+0.310	<0.1, strong H0

Table R2 (Table S2). Models controlling for training duration: extended models with both age and training duration. For each behavioural and neural metric, we report results on an extended model including both age at recording and training duration. In the extended models, the age term reflects the partial age effect controlling for training duration; the training term reflects any additional contribution of training duration beyond age.

R2.10.(Optional) Page 13, paragraph 3. Pairwise noise correlations.
As the authors write in the Discussion, changes in post-stimulus neural variability with aging may be related to changes in noise correlations (e.g., Miura et al, 2012). Given the rich population dataset of neural activity, the authors could test whether the results in this study are also related to changes in noise correlations during decision making. This is only a minor, optional suggestion.

We thank the reviewer for this thoughtful suggestion. As discussed in the manuscript, changes in single-neuron variability with aging may reflect multiple underlying biological mechanisms and can manifest across different neural scales, including pairwise noise correlations and broader population-level dynamics.

Indeed, we have been actively exploring noise correlations in this dataset and are currently developing these results as part of a separate manuscript dedicated to noise correlations during decision-making. We believe this will allow a more focused and in-depth treatment of both topics.

Thank you again for those insightful and constructive comments.

References

Churchland, Anne. K., Kiani, R., Chaudhuri, R., Wang, X.-J., Pouget, A., & Shadlen, M. N. (2011). Variance as a Signature of Neural Computations during Decision Making. *Neuron*, *69*(4), 818–831. https://doi.org/10.1016/j.neuron.2010.12.037

Hua, T., Li, X., He, L., Zhou, Y., Wang, Y., & Leventhal, A. G. (2006). Functional degradation of visual cortical cells in old cats. *Neurobiology of Aging*, *27*(1), 155–162. https://doi.org/10.1016/j.neurobiolaging.2004.11.012

International Brain Laboratory. (2020). *Behavior: Appendix 2: IBL protocol for mice training*. figshare. https://doi.org/10.6084/m9.figshare.11634729.v3

International Brain Laboratory. (2022a, May 4). *Appendix 2: IBL protocol for electrophysiology recording using Neuropixels probe* [Online resource]. figshare. Figshare. https://doi.org/10.6084/m9.figshare.19697896.v2

International Brain Laboratory, Aguillon-Rodriguez, V., Angelaki, D., Bayer, H., Bonacchi, N., Carandini, M., Cazettes, F., Chapuis, G., Churchland, A. K., Dan, Y., Dewitt, E., Faulkner, M., Forrest, H., Haetzel, L., Häusser, M., Hofer, S. B., Hu, F., Khanal, A., Krasniak, C., … Zador, A. M. (2021). Standardized and reproducible measurement of decision-making in mice. *eLife*, *10*, e63711. https://doi.org/10.7554/eLife.63711

International Brain Laboratory, Banga, K., Benson, J., Bhagat, J., Biderman, D., Birman, D., Bonacchi, N., Bruijns, S. A., Buchanan, K., Campbell, R. A. A., Carandini, M., Chapuis, G. A., Churchland, A. K., Davatolhagh, M. F., Lee, H. D., Faulkner, M., Gerçek, B., Hu, F., Huntenburg, J., … Zhang, Y. (2025). Reproducibility of in vivo electrophysiological measurements in mice. *eLife*, *13*, RP100840. https://doi.org/10.7554/eLife.100840

International Brain Laboratory, Benson, B., Benson, J., Birman, D., Bonacchi, N., Carandini, M., Catarino, J. A., Chapuis, G. A., Churchland, A. K., Dan, Y., Dayan, P., DeWitt, E. E., Engel, T. A., Fabbri, M., Faulkner, M., Fiete, I. R., Findling, C., Freitas-Silva, L., Gercek,

B., … Witten, I. B. (2025). A Brain-Wide Map of Neural Activity during Complex Behaviour. *Nature*, In press.

International Brain Laboratory, Bonacchi, N., Chapuis, G. A., Churchland, A. K., DeWitt, E. E. J., Faulkner, M., Harris, K. D., Huntenburg, J. M., Hunter, M., Laranjeira, I. C., Rossant, C., Sasaki, M., Schartner, M. M., Shen, S., Steinmetz, N. A., Walker, E. Y., West, S. J., Winter, O., & Wells, M. J. (2023). A modular architecture for organizing, processing and sharing neurophysiology data. *Nature Methods*, *20*(3), 403–407. https://doi.org/10.1038/s41592-022-01742-6

International Brain Laboratory, I. B. L. (2022b, May 4). *Video hardware and software for the International Brain Laboratory* [Online resource]. figshare. Figshare. https://doi.org/10.6084/m9.figshare.19694452.v1

Johnson, P. A., Nieuwenhuis, S., Mejías, J., & Urai, A. E. (2025). *A dynamical systems model of arousal-driven behavioural state transitions* (p. 2025.10.31.685593). bioRxiv. https://doi.org/10.1101/2025.10.31.685593

Liu, X., Zhou, Y., Liu, J., & Xu, G. (2025). Metformin improves age-related visual cortex dysfunction in mice by reducing noise correlation in the primary visual cortex. *Frontiers in Aging Neuroscience*, *17*. https://doi.org/10.3389/fnagi.2025.1572653

Wang, X., Zhang, B., Wang, H., Liu, J., Xu, G., & Zhou, Y. (2019). Aging affects correlation within the V1 neuronal population in rhesus monkeys. *Neurobiology of Aging*, *76*, 1–8. https://doi.org/10.1016/j.neurobiolaging.2018.11.025

Wulfse, F. (2023). *Explaining differences in learning speed in the decision-making task of the International Brain laboratory by light cycle and training time consistency* [Master's thesis]. Leiden University.

Yang, Y., Liang, Z., Li, G., Wang, Y., & Zhou, Y. (2009). Aging affects response variability of V1 and MT neurons in rhesus monkeys. *Brain Research*, *1274*, 21–27. https://doi.org/10.1016/j.brainres.2009.04.015

Dear editor and reviewers,

We thank you for the opportunity to revise our manuscript again and for the constructive feedback. We are encouraged that Reviewer 1 now supports publication and that Reviewer 2 found that most of the previous concerns had been addressed.

In this revision, we have focused specifically on the one remaining concern raised by Reviewer 2 regarding the potential contribution of behavioral variability to the modulation of neural activity. To address this point, we performed additional analyses of movement variability and revised the manuscript accordingly.

The changes in the manuscript include:

- We added new analyses to quantify variability of video-derived movement measures and assess age-related changes in movement variability (Figure 4 S7).
- We incorporated movement variability covariates into extended models to test whether the observed age effects persist after accounting for movement variability (Table S4).
- We summarized these additional analyses at the end of the Results (p. 12) and revised the Methods accordingly (p. 24).

Below, we provide a detailed response to the reviewer comments, with our replies in blue and with references to the revised manuscript and supplementary materials.

We believe that these additions further strengthen the manuscript. We hope that the revised manuscript is now suitable for publication in *Nature Communications*.

On behalf of all authors,
Fenying Zang and Anne Urai

Reply to reviewer #1

The authors have responded to all my concerns with detailed and thoughtful analyses. I now support publication. I thank the reviewers for the careful consideration of the issues raised.

We thank Reviewer 1 for the positive evaluation of our revised manuscript and for the thoughtful feedback throughout the review process. We are grateful that the reviewer now finds the manuscript suitable for publication.

Point-by-point reply to reviewer #2

I appreciate that the authors put a great effort into analyzing the movie data and generating additional figures to support their conclusions. The authors have addressed most of my previous comments. However, there is one remaining comment (and one minor change) that should be considered.

We thank Reviewer 2 for the careful evaluation of our revised manuscript and for the positive and constructive feedback. We are grateful that the reviewer found that most of the previous concerns had been addressed. Below, we respond to the remaining comment and minor point in detail.

Major comment

1. My previous comment 1 asked the following: “Was the modulation of activity merely a consequence of behavioral variability, or was it an effect of aging?” The authors mainly analyzed the “median” movement speed, which corresponded to Figure 1d. Although it is important to focus on the “median” speeds, I would suggest that the authors also consider the effects of behavioral “variability” on neural activity.

In Figure 1e and f, the authors analyzed reaction-time variability using the coefficient of variation (CV). How did the CV (or other measures of variability) of the DLC movements of the paw and nose-tip at pre- and post-stimulus in Figure 4 S6 correlate with aging?

In Table S3, in addition to using the median speeds, the authors could also use the CV of those speeds as a control to investigate whether the modulation of neural activity was merely a consequence of behavioral “variability” or an effect of aging.

By introducing movement CVs, the authors could also test the relationship between the change in movement variability between pre- and post-stimulus periods and Fano factor quenching in neurons. Another possible approach would be for the authors to subsample sessions and analyze neural activity only from sessions with similar movement variability but different ages.

We thank the reviewer for highlighting this important remaining concern. In the previous revision, we controlled for movement magnitude using median paw and nose-tip speed. Here, we extend that analysis by explicitly quantifying **movement variability**, to test whether the reported age-related neural effects could be accounted for by measured movement variability. Specifically, following the reviewer's suggestion, we performed two additional analyses, and a complementary follow-up analysis of the relationship between changes in movement variability and FF quenching.

First, building on our previous analyses of right-camera DLC movement, we quantified movement variability for the two representative features: right paw (task-relevant) and nose tip (task-irrelevant). From the speed traces, we quantified trial-to-trial movement variability using the coefficient of variation (CV), which captures variability relative to overall movement magnitude and aligns with the metric used in our main behavioral analyses, consistent with the reviewer's suggestion.

We first examined the corresponding time courses aligned to stimulus onset (Figure R1a,d). We then summarized movement variability within the same pre- and post-stimulus windows used to define our neural metrics. Across both measures, we did not observe consistent evidence for a systematic association between movement variability and age in either time window (Figure R1b–c,e–f). This provided a first descriptive characterization of movement variability across age, before we incorporated these measures as covariates in the extended models.

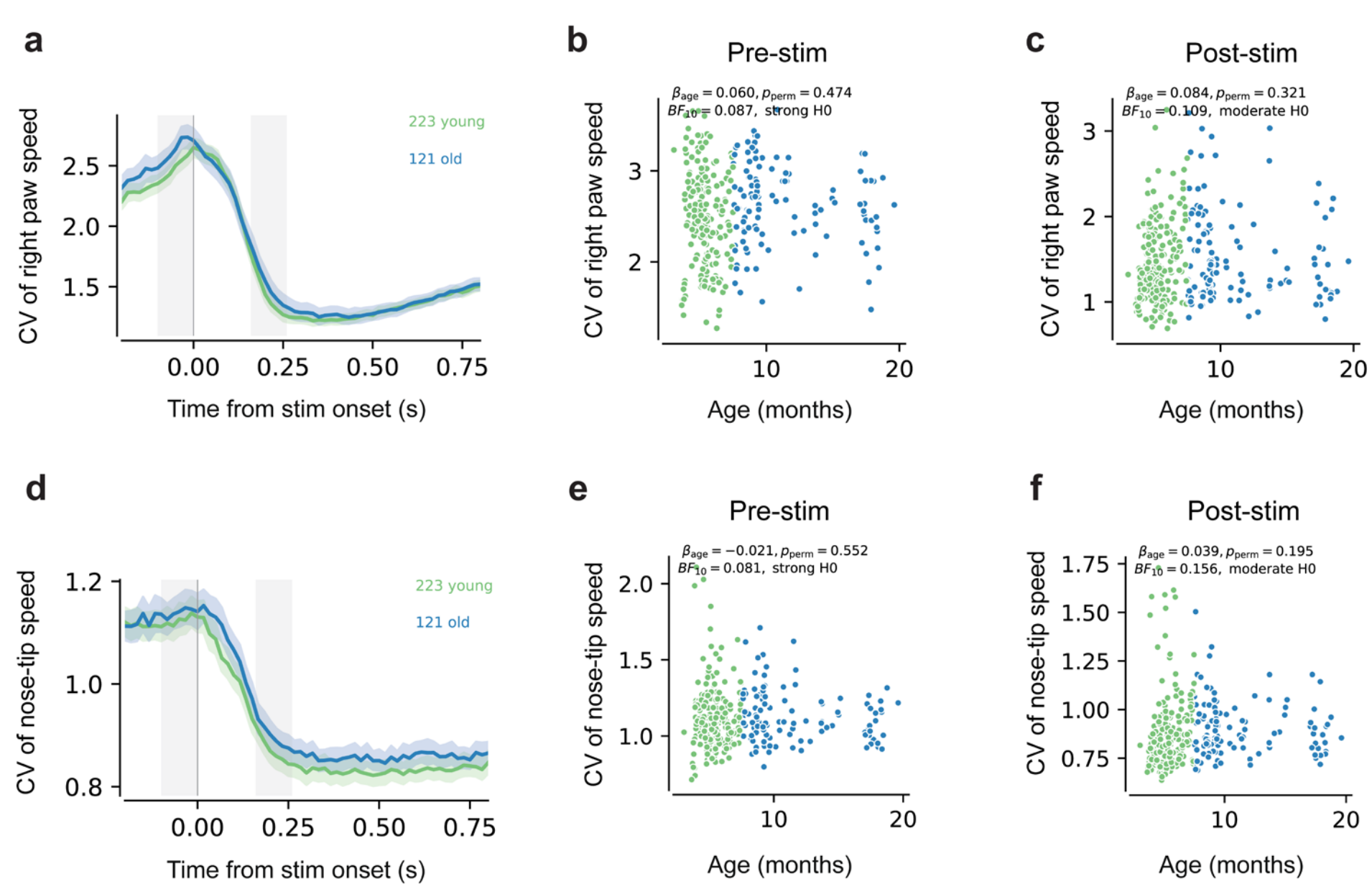

Figure R1 (Figure 4 S7). Right-camera DLC movement variability time courses and session-level summary metrics. (a) Time course of right paw speed variability, quantified as the coefficient of variation (CV) across trials and aligned to stimulus onset (t = 0), summarized across sessions (mean ± 95% confidence interval). Gray shaded regions indicate the pre-stimulus (−0.1 to 0 s) and post-stimulus (0.16 to 0.26 s) windows used to compute session-level movement variability summaries. **(b–c)** Session-wise

right paw speed CV within the pre-stimulus window (b) and post-stimulus window (c), plotted against mouse age; each dot corresponds to one session. **(d)** Time course of nose-tip speed variability, quantified as CV and aligned to stimulus onset, summarized across sessions (mean ± 95% confidence interval), with the same pre/post windows indicated. **(e–f)** Session-wise nose-tip speed CV within the pre-stimulus (e) and post-stimulus (f) windows versus age; each dot corresponds to one session.

Second, rather than subsampling sessions with similar movement variability, we retained all sessions and incorporated movement variability measures as covariates in the extended models, consistent with the approach used in our other control analyses. This allowed us to test the reviewer's concern while avoiding unnecessary loss of sample size. Specifically, for each neural metric, we fitted extended models that included age together with movement variability covariates, using the CV of right paw speed and nose-tip speed in the corresponding pre- and/or post-stimulus windows.

Overall, including movement variability covariates left our main age-related conclusions largely unchanged (Table R1). The qualitative age-related pattern was preserved for most neural metrics. For firing-rate contrast modulation, the evidence shifted from moderate evidence for H0 to weak evidence for H1 after including movement variability covariates, indicating weak evidence for an age effect in the adjusted model. For two additional metrics, the qualitative conclusion remained the same but the strength of evidence changed: for Fano factor quenching, evidence for H1 was attenuated from strong to weak, whereas for Fano factor contrast modulation, evidence for H1 increased from weak to strong. As in our previous movement-level control analysis, movement variability covariates also explained additional variance in several models.

metric	β_age	BF_evidence_age	β_paw_cv	BF10_paw_cv	β_nose_cv	BF10_nose_cv
pre_fr	+0.200	>100, strong H1	+0.020	3.07, moderate H1	-0.017	1.11, weak H1
post_fr	+0.207	>100, strong H1	+0.026	13.3, strong H1	+0.023	4.83, moderate H1
fr_delta_modulation	+0.552	6.59, moderate H1	+0.130	0.77, weak H0	-0.017	<0.1, strong H0
pre_ff	+0.014	<0.1, strong H0	+0.025	>100, strong H1	-0.009	0.28, moderate H0
post_ff	+0.066	>100, strong H1	+0.023	>100, strong H1	-0.010	0.42, weak H0
ff_quench	+0.046	2.89, weak H1	+0.006	>100, strong H1	-0.007	1.55, weak H1
ff_quench_modulation	-0.081	10.8, strong H1	-0.000	<0.1, strong H0	+0.007	<0.1, strong H0

Table R1 (Table S4). Additional controls using video-based movement variability from the right camera: extended model summary (Age + Paw CV+ Nose-tip CV). In sessions with QC-passing right-camera recordings (n = 343), we extracted right-paw and nose-tip speed variability, quantified as CV within the relevant pre- and/or post-stimulus windows, and added these as covariates in the extended models. The table reports β and BF_{10} for age, paw CV, and nose-tip CV terms.

As a final analysis prompted by the reviewer's suggestion, we also examined whether session-level changes in movement variability were related to FF quenching, because FF quenching captures the change in neural variability from the pre- to post-stimulus period. For each session, we computed the change in movement variability as Δ CV= post-stimulus CV− pre-stimulus CV for both right paw speed and nose-tip speed. We then examined the relationship between Δ

movement CV and FF quenching across sessions. We found evidence for an association for both measures, consistent with the extended-model results in Table R1. These results indicate that session-level changes in movement variability covary with FF quenching, but do not by themselves explain the broader pattern of age-related neural effects observed in our data. We believe this is an exciting additional phenomenon that we are planning to explore in future theoretical work.

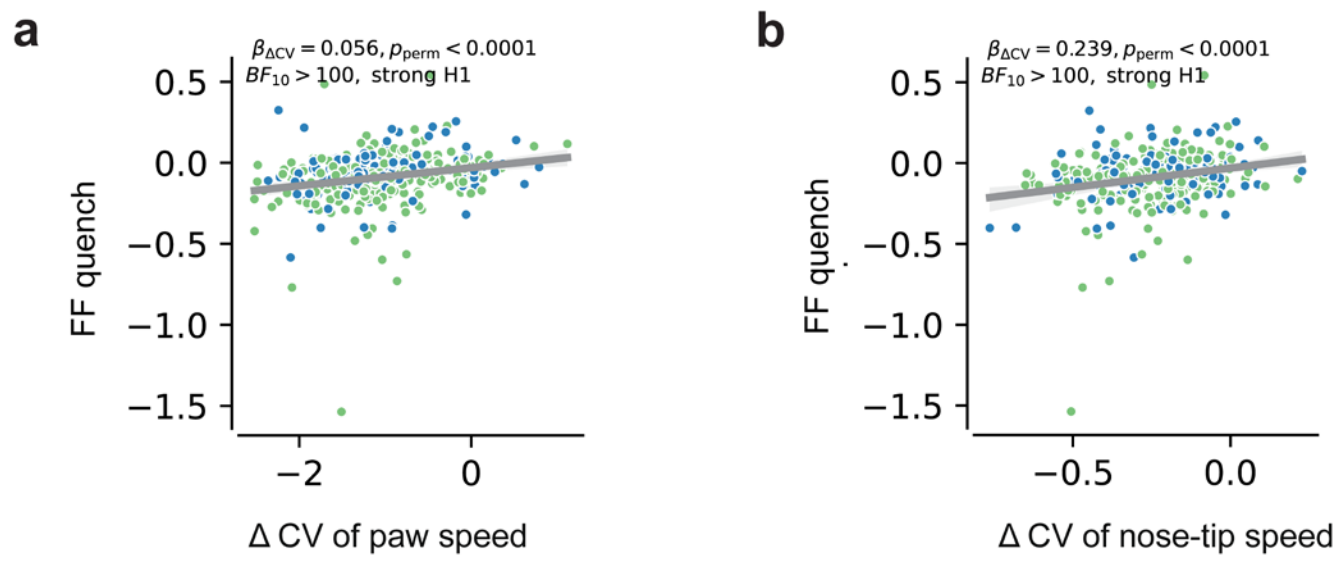

Figure R2. Relationship between FF quenching and change in movement variability across sessions. (a) Right paw. Session-level FF quenching plotted against the change in right-paw speed variability, quantified as Δ CV= post-stimulus CV− pre-stimulus CV. **(b)** Nose tip. Session-level FF quenching plotted against the corresponding change in nose-tip speed variability. Each dot represents one session.

Together, these analyses indicate that the reported age-related differences in firing rate and neural variability are unlikely to be explained solely by the measured movement variability captured by these video-derived covariates. In our data, including session-level movement variability in the models did not account for the overall pattern of age-related neural effects. **At the same time, these analyses do not rule out the possibility that behavioral variability may still contribute mechanistically to age-related neural changes. Importantly, the movement measures considered here were aggregated at the session level and included as covariates, which is useful for assessing potential confounding but is not sufficient to fully resolve the structure of behavioral-neural coupling. Addressing this question will require more targeted analyses and richer models that relate behavioral and neural variability more directly, which we plan to pursue in future work. We thank the reviewer again for this helpful suggestion.**

In the revised manuscript, we now added those analyses to the section discussing movement and other potential contributors to the observed age effects (Results, p. 12, lines 21–32).

> “To further test whether the age-related neural effects could be accounted for by movement variability beyond average movement speed, we performed an additional control analysis using the coefficient of variation (CV) in trial-to-trial right-paw and nose-tip speed within the same pre- and post-stimulus windows. Session-level movement variability did not show consistent evidence for a systematic association with age, across measures and time windows (Figure 4-S7). Although these covariates explained additional variance in several cases, adding these movement-variability measures as covariates to the extended models left the main age-related pattern broadly unchanged (Table S4).

Together, these analyses suggest that while video-derived movement explains additional variance in firing-rate and variability metrics, the reported age-related neural effects persist after accounting for both movement magnitude and movement variability."

We revised the Methods accordingly (p. 24, lines 20–40):

"...We summarized movement by computing median speed within the same pre- and post-stimulus windows used for neural metrics (pre: −0.1 to 0 s; post: 0.16 to 0.26 s), yielding pre- and post-stimulus movement measures for both paw and nose tip. To quantify movement variability, we additionally computed the coefficient of variation (CV; standard deviation / mean) in trial-to-trial paw and nose-tip speed within the same pre- and post-stimulus windows.

Sessions were required to pass IBL extended quality control for the right camera (videoRight and dlcRight). This yielded n = 343 sessions (out of total 367 sessions) with valid right-camera movement measures. We then fitted extended statistical models for each neural metric by adding either movement-speed covariates or movement-variability covariates to the age-only models. Movement covariates were matched to each neural metric by time window and contrast structure. Metrics defined in a single window used the corresponding pre- or post-stimulus movement measure, whereas metrics spanning both windows included both pre- and post-stimulus covariates. For neural metrics retaining contrast-specific values (pre- and post-stimulus firing rate), movement measures were computed separately for each session and contrast condition; for neural metrics not retaining contrast (contrast modulation of firing rate, pre- and post-stimulus Fano Factor, Fano Factor quench, and contrast modulation of Fano Factor), movement measures were then aggregated across contrast conditions to yield one value per session, window, and feature. We applied the same matching scheme in the movement-variability control models."

Minor comment

Caption of Figure 4 S6: median ± 95% CI.
I think "CI" appears here for the first time. Please write it as "confidence interval".

We thank the reviewer for pointing this out. We have revised the caption of Figure 4 S6.

Reviewer #2 (Remarks on code availability):

The code is well organized. It provides a README file with enough instructions.

We thank Reviewer 2 for the positive assessment of the code availability and documentation.
